# Comprehensive phenotypic analysis of knockout mice deficient in cyclin G1 and cyclin G2

**DOI:** 10.1038/srep39091

**Published:** 2016-12-16

**Authors:** Shouichi Ohno, Jun-ichiro Ikeda, Yoko Naito, Daisuke Okuzaki, Towa Sasakura, Kohshiro Fukushima, Yukihiro Nishikawa, Kaori Ota, Yorika Kato, Mian Wang, Kosuke Torigata, Takashi Kasama, Toshihiro Uchihashi, Daisaku Miura, Norikazu Yabuta, Eiichi Morii, Hiroshi Nojima

**Affiliations:** 1 Department of Molecular Genetics, Research Institute for Microbial Diseases, Osaka University, 3-1 Yamadaoka, Suita, Osaka 565-0871, Japan; 2 Department of Pathology, Graduate School of Medicine, Osaka University, 1-1 Yamadaoka, Suita, Osaka 565-0871, Japan; 3 First Department of Oral and Maxillofacial Surgery, Graduate School of Dentistry, Osaka University, 1-8 Yamadaoka, Suita, Osaka 565-0871, Japan; 4 Department of Pharmacy, Hyogo University of Health Sciences, Kobe 650-8530, Japan

## Abstract

Cyclin G1 (CycG1) and Cyclin G2 (CycG2) play similar roles during the DNA damage response (DDR), but their detailed roles remain elusive. To investigate their distinct roles, we generated knockout mice deficient in CycG1 (G1KO) or CycG2 (G2KO), as well as double knockout mice (DKO) deficient in both proteins. All knockouts developed normally and were fertile. Generation of mouse embryonic fibroblasts (MEFs) from these mice revealed that G2KO MEFs, but not G1KO or DKO MEFs, were resistant to DNA damage insults caused by camptothecin and ionizing radiation (IR) and underwent cell cycle arrest. CycG2, but not CycG1, co-localized with γH2AX foci in the nucleus after γ-IR, and γH2AX-mediated DNA repair and dephosphorylation of CHK2 were delayed in G2KO MEFs. H2AX associated with CycG1, CycG2, and protein phosphatase 2A (PP2A), suggesting that γH2AX affects the function of PP2A via direct interaction with its B’γ subunit. Furthermore, expression of CycG2, but not CycG1, was abnormal in various cancer cell lines. Kaplan–Meier curves based on TCGA data disclosed that head and neck cancer patients with reduced CycG2 expression have poorer clinical prognoses. Taken together, our data suggest that reduced CycG2 expression could be useful as a novel prognostic marker of cancer.

Cyclin G1 (CycG1), a member of the cyclin family^1^, recruits protein phosphatase 2A (PP2A) to its targets by interacting with the PP2A_B’γ subunit and regulating PP2A function^2^. Expression of CycG1, which is a transcriptional target of the tumor suppressor p53, is induced during the DNA damage response (DDR)^3^. In turn, CycG1 regulates the stability of p53 protein via dephosphorylation of MDM2, the ubiquitin ligase for p53, at T216 (MDM2-pT216), thus constituting a negative feedback system that attenuates p53 activity^4^,^5^. At the initial stage of DNA damage, CycG1 interacts directly with MDM2 and promotes formation of the ARF/MDM2 complex, but the CycG1–MDM2 complex dissociates from ARF and subsequently captures B’γ^6^. Thus, the main role of CycG1 is to mediate or regulate the function of p53 in the nucleolus^6^,^7^. Although CycG1-deficient (*Ccng1*^−/−^) mice are born and mature normally, they are more sensitive to γ-radiation than wild-type (WT) mice^3^. In some cells, CycG1 exerts positive effects on cell growth: exogenously overexpressed CycG1 promotes cancer cell proliferation^3^,^5^,^8^, whereas antisense-mediated down-regulation of CycG1 inhibits cell growth^9^,^10^,^11^. In other cells, however, CycG1 negatively influences proliferation: in various systems, overexpression of CycG1 can induce G1-phase arrest^7^,^12^, G2/M arrest^13^, or apoptotic cell death^14^,^15^. In cancer patients, overexpression of the CycG1 gene (*CCNG1*) is frequently observed in osteosarcoma^16^, breast and prostate cancer^17^, colorectal cancer^18^, and hepatocellular carcinoma^19^. CycG1 is the target of the liver-specific micro-RNA miR-122^20^,^21^; in human hepatoma cells, alcohol facilitates hepatitis C virus RNA replication by upregulating miR-122 expression and thereby inhibiting production of cyclin G1^22^. Notably, overexpression of CycG1 promotes the epithelial–mesenchymal transition (EMT) and metastasis by enhancing AKT activation, leading to stabilization of SNAIL, a critical mediator of the EMT^23^.

Cyclin G2 (CycG2), a CycG1 homolog^24^, also recruits the PP2A holoenzyme via association with B’γ^25^. CycG2 targets Chk2-pT68 for dephosphorylation^26^ by recruiting the PP2A complex to DNA repair foci, and CycG2-deficient cells exhibit delayed dephosphorylation of Chk2-pT68^27^. CycG2 also cooperates with B’γ to dephosphorylate γH2AX, another DNA repair factor, as demonstrated by the observation that dephosphorylation of γH2AX-pS139 is delayed in CycG2-deficient or siRNA-mediated B’γ knockdown cells^27^. Ectopic expression of CycG2 inhibits cell growth by inducing cell-cycle arrest^25^,^28^,^29^,^30^,^31^. CycG2 expression is regulated by p63, a homolog of p53, which contributes to G2/M arrest during the DDR^32^. CycG2 expression correlates with cell-cycle inhibition and is upregulated in response to diverse growth inhibitory stimuli including hypoxia and endoplasmic reticulum stress; however, it is repressed by mitogenic signals^33^,^34^,^35^. Nodal signaling promotes transcription of the human CycG2 gene (*CCNG2*) by upregulating the forkhead transcription factor FoxO3a^31^,^36^. Moreover, *CCNG2* is a primary target of estrogen receptor in MCF-7 cells^37^. The CycG2 mRNA level is high at G0 phase, declines as cells enter the cell cycle, and increases again from mid-S to early G2 phase^38^. Notably, CycG2 expression is down-regulated in several cancers, including thyroid and oral cancers^29^,^39^. By contrast, upon antibody-mediated inhibition of HER2 signaling, CycG2 is upregulated and translocates to the nucleus^30^. Based on these observations, CycG1 and CycG2 may serve as prognostic biomarkers and therapeutic targets. However, the precise functions of CycG1 and CycG2 in cancer cells are not fully understood.

In this study, we sought to investigate the distinct roles of CycG1 and CycG2 by generating mice deficient in CycG1 (G1KO) or CycG2 (G2KO), as well as double knockout (DKO) mice deficient in both CycG1 and CycG2. We found only one abnormal phenotype in the knockout mice, i.e., reduced incidence of diethylnitrosamine (DEN)-induced hepatocarcinogenesis. G2KO-derived mouse embryonic fibroblasts (MEFs), but not G1KO or DKO MEFs, were resistant to DNA damage caused by camptothecin and ionizing radiation (IR), and underwent G2/M arrest. We discuss the origins of these phenotypes, and propose that reduced CycG2 expression may serve as a novel prognostic marker of cancer.

## Results

### Generation of *Ccng1* ^−/−^ *Ccng2* ^−/−^ DKO mice

To understand the physiological role of CycG1 and CycG2 in more detail, we generated *Ccng1*^−/−^*Ccng2*^−/−^ double knockout (DKO) mice. Previously, we generated *Ccng1*^−/−^ (G1KO) mice by disrupting the second exon of *Ccng1*^3^. For this study, to avoid generation of partial CycG1 mRNA by alternative splicing, we generated another version of the G1KO mouse by removing all protein coding exons of *Ccng1* (Supplementary Fig. S1a). Heterozygous *Ccng1*^+/−^ *Ccng2*^+/−^ mutant mice were obtained by crossing chimeric G1KO mice with *Ccng2*^−/−^ (G2KO) mice^27^, and homozygous DKO mice were obtained by mating these heterozygous mice; the genotypes of these offspring mice were analyzed by RT-PCR of genomic DNA isolated from tail snips (Supplementary Fig. S1b–d). G1KO, G2KO, and DKO mice were all born and matured normally, and exhibited normal fertility. When *Ccng1*^+/−^ (Supplementary Table S1a) or *Ccng2*^+/−^ (Supplementary Table S1b) mice were crossed separately, they generated similar numbers of male/female offspring irrespective of *Ccng1* or *Ccng2* genotype. By contrast, when *Ccng1*^+/−^ *Ccng2*^+/−^ mice were crossed, they generated fewer offspring with the G2KO genotype than the G1KO or WT genotype (Supplementary Table S1c); only 4 of 214 offspring had the DKO genotype. These mice were maintained for up to 30 months and exhibited no evidence of illness or spontaneous carcinogenesis, suggesting that the absence of both *Ccng1* and *Ccng2* does not increase the incidence of diseases, including cancer (Supplementary Table S2).

### G2KO MEFs, but not WT, G1KO, or DKO MEFs, are resistant to DSB insults

**Figure 1:**
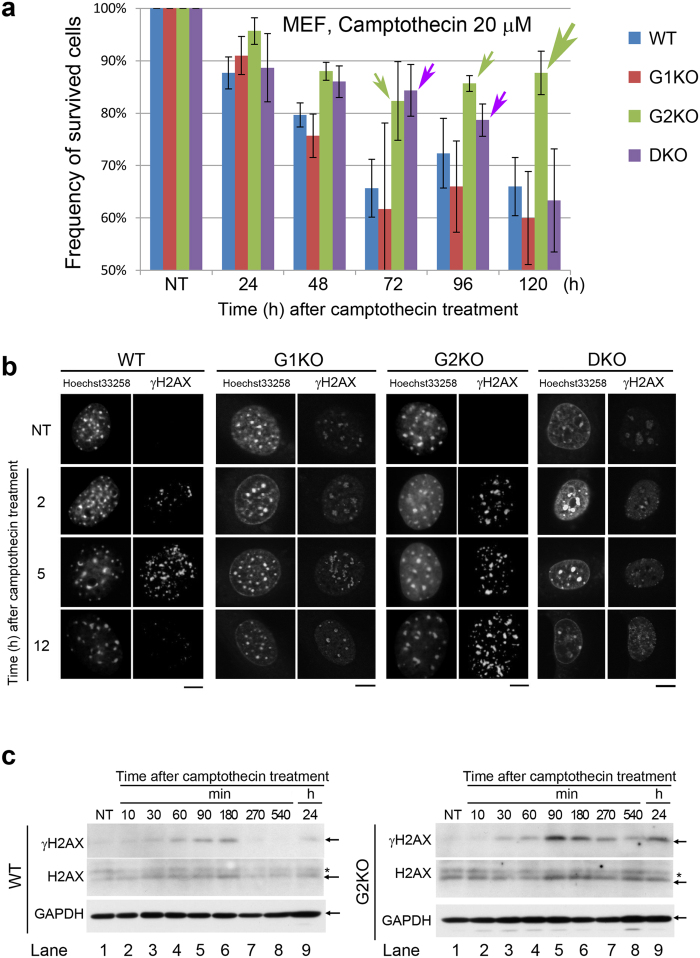
G2KO MEFs, but not WT, G1KO, or DKO MEFs, are resistant to camptothecin. (**a**) Bar graphs represent survival rate for WT, G1KO, G2KO, and DKO MEFs treated for the indicated times (h) with 20 μM camptothecin. Green arrows indicate that more than 80% of G2KO MEFs survived at 72, 96, and 120 h. Purple arrows indicate that nearly 80% of DKO MEFs survived at 72 and 96 h. NT, non-treated. (**b**) Typical images of WT, G1KO, G2KO and DKO MEFs treated for the indicated times (h) with 20 μM camptothecin, and then stained with Hoechst 33258 (DNA) and anti-γH2AX antibody (H2AX- pS139). Bar, 10 μm. (**c**) Western blot to detect H2AX, γH2AX, and GAPDH (loading control) in WT and G2KO MEFs treated for the indicated times (h) with 20 μM camptothecin. Arrows indicate H2AX and γH2AX bands. Asterisks denote non-specific bands.

Because CycG1 and CycG2 are involved in the DDR, we tested whether loss of CycG1 and CycG2 would result in a distinct response to 20 μM camptothecin (Fig. S2), a chemical carcinogen that generates double-stranded breaks (DSBs), in MEFs derived from the knockout mice (Supplementary Fig. S1e–g). G1KO and WT MEFs that were treated with camptothecin for 72–96 h had lower survival rates than G2KO and DKO MEFs (Fig. 1a). By contrast, G2KO MEFs treated with 20 μM camptothecin for 120 h retained a higher frequency of surviving cells than WT, G1KO, and DKO MEFs; the green arrows in Fig. 1a indicate that more than 80% of G2KO MEFs survived at 72, 96, and 120 h. Moreover, the purple arrows also indicate that nearly 80% of DKO MEFs survived at 72 and 96 h, suggesting that DKO was more resistant to camptothecin than WT and G1KO, but less resistant than G2KO.

We previously showed that CycG2, but not CycG1, co-localizes with γH2AX foci in the nucleus after γ-IR, suggesting that CycG2 is recruited to sites of DNA repair^27^. To determine whether camptothecin treatment would also generate new γH2AX foci, we immunostained MEFs with anti-γH2AX antibody. Foci were observed in WT, G1KO, G2KO, and DKO MEFs treated with 20 μM camptothecin for 2, 5 and 12 h (Fig. 1b). In G2KO MEFs, but not in WT or G1KO MEFs, the foci were detected even at 12 h, suggesting that DNA repair was delayed in G2KO relative to the other two strains (Fig. 1b). Only a few faint γH2AX signals were observed in DKO MEFs, suggesting that either CycG1 or CycG2 is required for generation of γH2AX foci. Western blots also revealed abundant γH2AX in G2KO MEFs even after 270 min of camptothecin treatment, by which time the γH2AX band had almost disappeared in WT MEFs, confirming that γH2AX-mediated DNA repair was delayed in G2KO MEFs (Fig. 1c). These observations suggest that γH2AX expression persists in G2KO MEFs because these cells have a greater burden of DNA damage.

### G2KO MEFs, but not WT, G1KO, or DKO MEFs, enter cell cycle arrest after γ-IR

**Figure 2:**
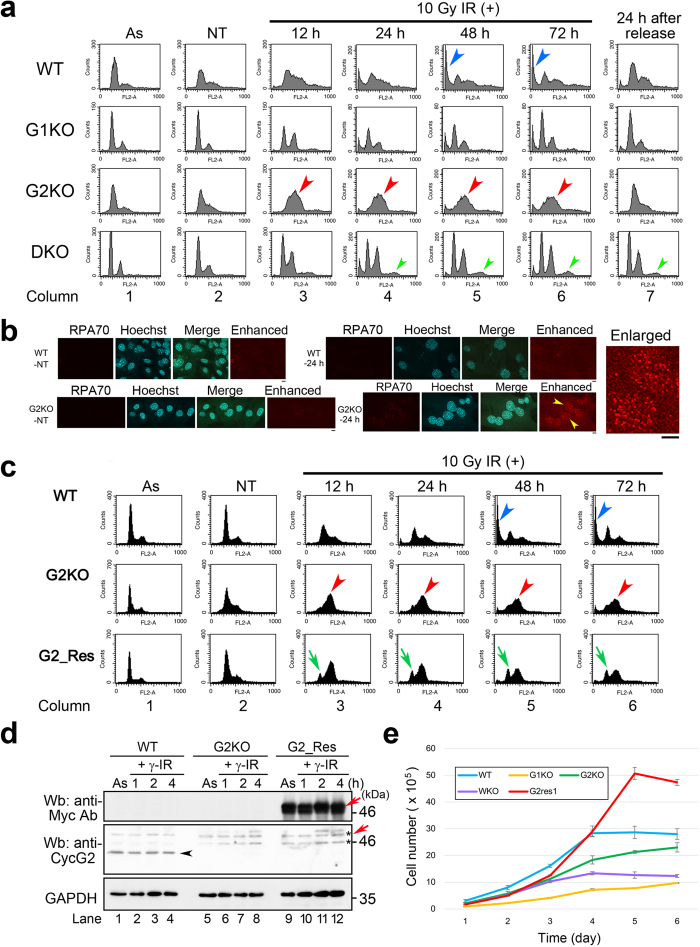
G2KO MEFs, but not G1KO or DKO MEFs, arrest at S-G2/M phase after 10 Gy γ-radiation. (**a**) Flow cytometry analysis of WT, G1KO, G2KO and DKO MEFs that were collected after thymidine–aphidicolin double block and treated with 10 Gy γ-IR for the indicated times (h); cells were stained with propidium iodide (PI), and cell-cycle profiles were determined by flow cytometry. The profiles of these MEFs 24 h after release from the double thymidine block without γ-IR are also shown in the rightmost columns. Red or green arrowheads indicate the delayed G2/M phase peak or extra peak putatively derived from endoreplication, respectively. As, asynchronous growth. NT, non-treated. (**b**) Typical IF images of WT and G2KO MEFs, immunostained with an anti-RPA70 antibody at 24 h after treatment with and without (NT) 10 Gy γ-IR treatment. Hoechst33258 was used to stain the nuclear DNA. “Enhanced” means the contrast-enhanced image for RPA70-immunostaining. The rightmost panel shows enlarged images of nuclear RPA70 dots in cells indicated by yellow arrowheads. (**c**) Flow cytometry analysis of WT MEFs, G2KO MEFs, and G2KO MEFs expressing Myc-CycG2 (G2_Res). Blue arrowheads (WT), red arrowheads (G2KO), and green arrows (G2_Res) indicate Sub-G1, G2/M and G1 phase peaks, respectively. (**d**) Western blot analysis using anti-Myc and anti-CycG2 antibodies suggested the physiological level of Myc-CycG2 expression (red arrows) did not surpass that of endogenous CycG2 (black arrowhead). Asterisk indicated a putative non-specific band. (**e**) Growth curves show that G1KO, G2KO and DKO MEFs grew slower than WT MRFs, whereas G2_Res MEFs grew much faster than WT MEFs.

Next, we examined the cell-cycle progression profiles of these MEFs by flow cytometry. For this purpose, we synchronized the cell cycle by thymidine–aphidicolin double block (TADB) and treated the cells with or without IR using 10 Gy γ-ray (Fig. 2). MEFs not subjected to TADB are shown in column 1 (asynchronous; As). MEFs not subjected to γ-IR treatment at 0 or 24 h after TADB are shown in columns 2 (non-treated; NT) and 7 (24 h after release), respectively. Relative to these controls, WT and G1KO cells exhibited significant increases in the sub-G1 population (an indicator of apoptotic cell death) 48 and 72 h after γ-IR (blue arrowheads in Fig. 2a). By contrast, G2KO and DKO MEFs exhibited no such increase in the sub-G1 population. Bar graph presentation of the data (Fig. S3) confirms these phenotypes. In all cell lines, sensitivity to γ-IR reflected the response to camptothecin treatment (Fig. 1a).

Flow cytometry also revealed that G2KO MEFs underwent a marked cell-cycle arrest following γ-IR (red arrowheads in Fig. 2a), which appears to have occurred in G2/M phase as judged by the increase in the G2/M peak in the histogram of G2KO MEFs (Fig. S3). Given that the cell-cycle profile of G2KO MEFs 24 h after TADB release (column 7) was similar to that of NT, this phenotype was primarily due to γ-IR treatment (column 1). Because it is generally difficult to identify an S phase population by flow cytometry, we immunostained RPA70, a DNA replication regulator whose level changes during the cell cycle, to monitor S phase progression. We found that nuclear RPA70 signals were present in most G2KO MEFs at 24 h after γ-IR; by contrast, no such RPA70 signals were detected in WT MEFs and non-treated MEFs (Fig. 2b). These results suggest that γ-IR caused a delay in S phase in G2KO MEFs, activated a checkpoint machinery resulting in cell cycle arrest in G2/M phase.

The cell cycle arrest phenotype in G2KO MEFs was also observed using two distinct synchronization methods, single thymidine block (Fig. S4a) and double thymidine block (Fig. S4b and c). By contrast, MEFs of the other strains arrested at G1/S in response to TADB (columns 2–7). Notably, DKO exhibited an S-phase delay and an extra peak at 12–72 h following γ-IR (green arrowheads in Fig. 2a), suggesting endoreplication. We plan a detailed study of this phenotype in future work.

Exogenous expression of Myc-CycG2 in G2KO MEFs (G2_Res) partially rescued this G2/M arrest (Fig. 2c); this was reflected by the appearance of G1-phase peaks (green arrows in Fig. 2c). Western blot analysis using anti-Myc and anti-CycG2 antibodies suggested the physiologic level of Myc-CycG2 expression, based on its band intensity (red arrow in Fig. 2d), did not surpass that of endogenous CycG2 (black arrowhead in Fig. 2d), although the Myc-CycG2 band was hidden behind a putative non-specific band (asterisk in Fig. 2d). Growth curves show that G1KO, G2KO, and DKO MEFs grew slower than WT MEFs, whereas G2_Res MEFs grew much faster (Fig. 2e), consistent with successful expression of CycG2, which promotes cell growth in G2_Res MEFs. These results suggest that CycG2 is required for proper progression through G2/M phase of the cell cycle.

Moreover, G2KO mice also tended to have a higher survival rate following γ-IR treatment, as evaluated by log-rank test of Kaplan–Meier survival curves (Supplementary Fig. S5a), although neither Cochran–Mantel–Haenszel (CMH) values (*P* = 0.145) nor Gehan-Breslow-Wilcoxon (GBW) values (*P* = 0.069) were statistically significant. When we performed immunohistochemistry for active caspase-3 in the small intestine, one of the organs most sensitive to IR, we found neither histological differences nor elevated caspase-3 staining in these mice (Supplementary Fig. S5b).

### Western blot analysis of G2KO MEFs after γ-IR

**Figure 3:**
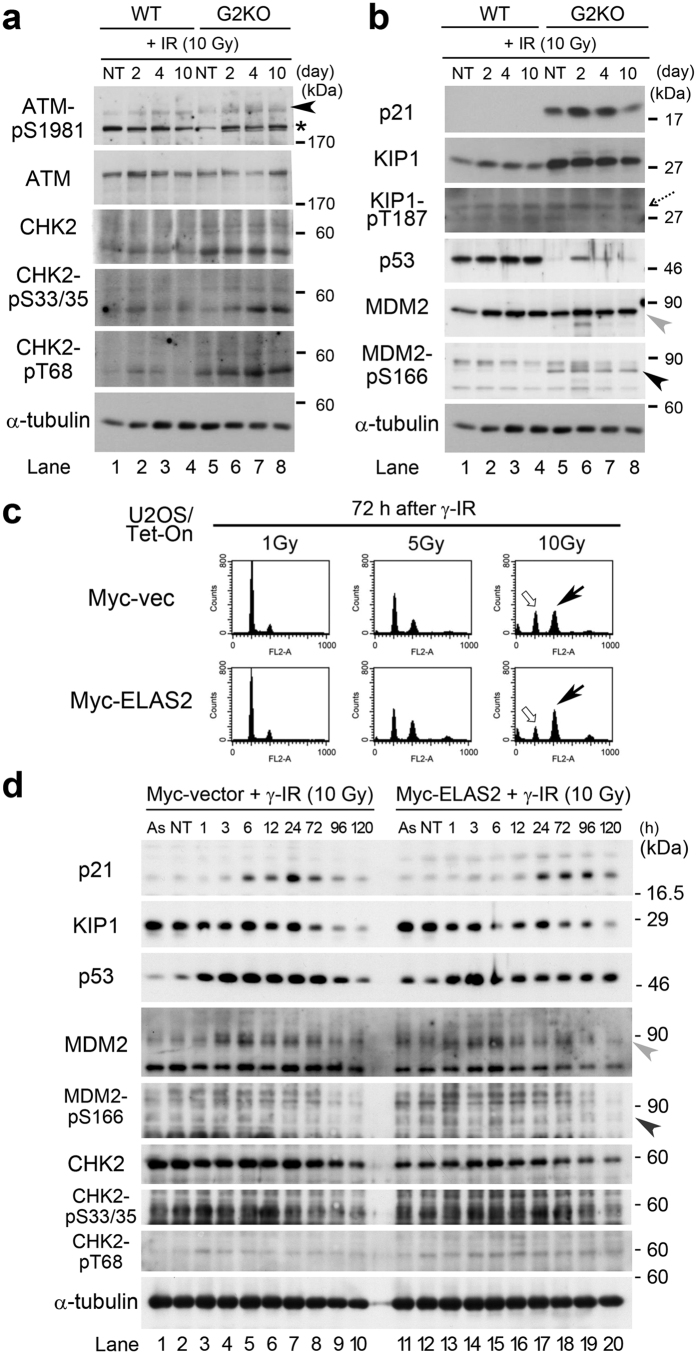
Western blot analysis on WT and G2KO MEFs after 10 Gy γ-radiation. (**a,b**) WT and G2KO MEFs in their logarithmic growth phase were irradiated by γ-IR (10 Gy). The cell extracts were collected at the indicted times and subjected to western blot analysis with (**a**) anti-ATM-pS1981, anti-ATM, anti-CHK2, anti-CHK2-pS33/55, or anti-CHK2-pT68, and (**b**) anti-p21, anti-p27 (KIP1), anti-KIP1-pT187, anti-p53, anti-MDM2 or anti-MDM2-p5166 antibody. α-tubulin was detected as a loading control. (**a**) Arrowhead, and (**b**) dotted arrow, grey arrowhead, and tilted black arrowhead indicate bands representing ATM-pS1981, KIP1-pT187, MDM2 and MDM2-p5166, respectively. Asterisks denote non-specific bands. NT, non-treated. (**c**) Flow cytometry analysis showing that exogenous expression of ELAS2 in U2OS cells induces G2/M arrest after γ-IR treatment. U2OS/Tet-On cells stably expressing Myc-ELAS2 or vector alone (Myc-vec) were treated with 10 Gy γ-IR for the indicated times (h) in the presence of doxycycline (Dox). Cells were stained with propidium iodide (PI), and cell-cycle profiles were determined by flow cytometry. Percentages indicate the sub-G1 population. (**d**) Western blot to detect p21, KIP1, p53, MDM2, MDM2-p5166, CHK2, CHK2-pS33/3, CHK2-pT68, and α-tubulin (loading control) in U2OS/Tet-On cells stably expressing Myc-ELAS2 or vector alone (Myc-vec) that were treated with 10 Gy γ-IR for the indicated times (h). As, asynchronous growth. NT, non-treated.

Previously, we showed that the CycG2-B’γ complex promotes dephosphorylation of CHK2-pT68 and that loss of CycG2 delays activation of the damage checkpoint machinery^27^. Consistent with this, in WT MEFs, CHK2-pT68 band intensity peaked at 2 h (lane 2 in Fig. 3a), and then gradually decreased at 4 (lane 3) and 10 h (lane 4) after γ-IR, whereas in G2KO MEFs the band remained strong even 10 h after IR (lanes 5–8 of CHK2-pT68 panel in Fig. 3a). Interestingly, a similar profile was also observed for CHK2-pS33/35, suggesting that the CycG2–B’γ complex promotes dephosphorylation of CHK2-pS33/35 *in vivo*. Notably, CHK2 band intensity was also elevated in G2KO MEFs relative to WT MEFs, suggesting that CHK2 is stabilized and/or upregulated in G2KO MEFs.

The most conspicuous difference between WT and G2KO MEFs was the level of p21, an inhibitor of cyclin dependent kinase (CDK) that regulates cell-cycle progression. The p21 band appeared in G2KO MEFs, but not in WT MEFs, peaking at 2–4 h and then diminishing 10 h after γ-IR (Fig. 3b). Because p21 inhibits cell-cycle progression, this observation suggests that an elevated p21 protein level plays a role in the G2/M arrest of G2KO MEFs after γ-IR. Moreover, the protein level of Kip1, another CDK inhibitor, is also elevated in G2KO MEFs relative to WT MEFs, although dephosphorylation of Kip1 was not altered (dotted arrow in Fig. 3b). Given that p21 inhibits cell-cycle progression, the increase in the p21 level may also play a role in G2/M arrest of G2KO MEF after γ-IR. These results suggest that loss of CycG2 results in abnormal processing of DNA damage regulators.

The reduced response to p21 and KIP1 induction after γ-IR in WT MEFs was not due to a lack of p53 because the p53 level was constitutively high in WT MEFs, in contrast to G2KO MEFs where p53 was induced sharply at 2 h after γ-IR than G2KO MEFs (Fig. 3b). Moreover, the level of MDM2, an E3 ubiquitin ligase involved in the degradation of p53 by the proteasome, was similar between WT and G2KO MEFs (Fig. 3b). Because the MDM2 gene is a transcriptional target of p53, the p53-MDM2 axis may be normal in these MEFs. Nonetheless, only G2KO MEFs showed an extra band when probed with an anti-MDM2-pS166 antibody, suggesting that the regulation of MDM2-pS166 may be abnormal in G2KO MEFs (arrowhead in Fig. 3b). Considering the role of CycG1-B’γ complex as a regulator of MDM2-pT216 dephosphorylation^5^, our results suggest that CycG2-B’γ complex may also have a role in the dephosphorylation of MDM2-pT166. Further studies will be required to uncover the details of the mechanisms involved.

### G2/M arrest is caused by inhibition of CycG2 function

We next investigated whether G2/M arrest of G2KO MEFs after 10 Gy γ-IR would also occur when CycG2 function was inhibited. Previously, we showed that the ELAS2 peptide inhibits CycG2 function by blocking formation of the CycG2-B’γ complex^40^. Indeed, flow cytometry showed that the ratio between the G2/M peak (black arrow) and G1 peak (white arrow) was higher in Myc-ELAS2–expressing U2OS cells than in Myc-vector–expressing cells (Fig. 3c). Expression of Myc-ELAS2 and Myc-vector were induced by addition of doxycycline (Dox) into the medium. This G2/M arrest was not observed after 1 or 5 Gy γ-IR (Fig. 3c), suggesting that a minimum level of DSBs (induced by 10 Gy γ-IR) is required for G2/M arrest. In a western blot analysis, the band intensity for CHK2-pT68 peaked at 1 h after γ-IR and gradually decreased afterwards in Myc-vector–expressing cells, whereas in Myc-ELAS2–expressing cells, the band intensity remained strong up to 120 h after γ-IR (Fig. 3d). By contrast, band intensities for p53, MDM2, CHK2 and CHK2-pS33/35 did not differ significantly between Myc-vector– and Myc-ELAS2–expressing cells, suggesting that these proteins play a minimal role in G2/M arrest in this context. Moreover, the p21 protein increased in both cell types, peaking at 24 h and 72–96 h after γ-IR in Myc-vector– and Myc-ELAS2–expressing cells, respectively (Fig. 3d). Because the p21 level also increased after γ-IR in Myc-vector–expressing cells that did not undergo G2/M arrest, this result indicates that elevation of the p21 level is not directly involved in this arrest. We observed no apparent difference in KIP1 band intensity. These results suggest that G2/M arrest in cells deficient in CycG2 is mediated by damage checkpoint machinery induced by persistent phosphorylation of CHK2 at T68.

### CycG2 is required for formation of the complex between H2AX and PP2A C after γ-IR

**Figure 4:**
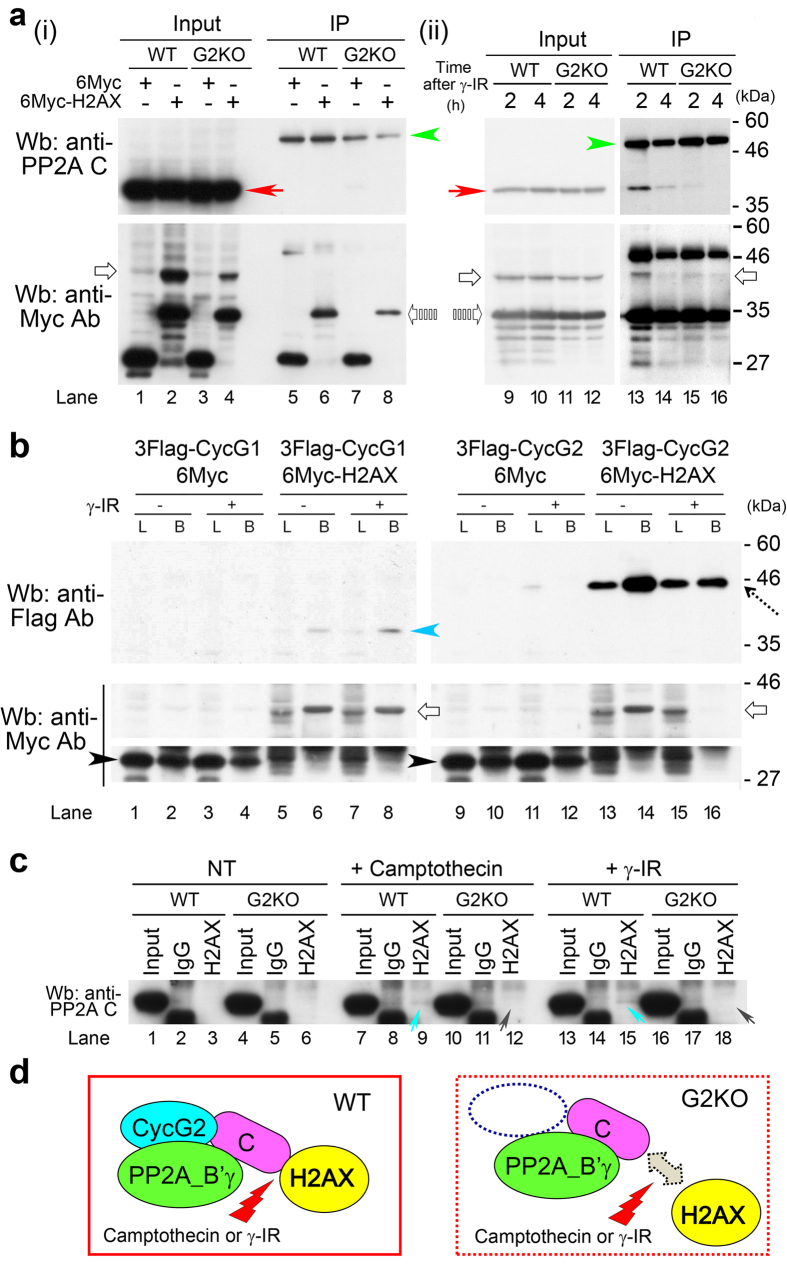
H2AX associates with CycG1, CycG2, and PP2A *in vivo*. (**a**) Western blot analyses were performed on extracts from WT or G2KO MEFs expressing 6Myc-vector or 6Myc-H2AX in the absence (i) or presence (ii) of γ-IR treatment. Cell extracts (input) or immunoprecipitated cell extracts (IP) were analyzed using anti-PP2A C subunit or anti-Myc antibody. Red arrows, white arrows, dotted white arrows, or green arrowheads indicate a band for PP2A C subunit, 6Myc-H2AX, a putative truncated form of 6Myc-H2AX, or immunoglobulin heavy chain proteins, respectively. (**b**) Western blot analyses were performed on extracts from HEK-293T cells expressing 3FLAG-CycG1+6Myc vector, 3FLAG-CycG1+6Myc-H2AX, 3FLAG-CycG2+6Myc vector, or 3FLAG-CycG2+6Myc-H2AX, respectively, in the absence (−) or presence (+) of γ-IR treatment. Blue arrowhead, white arrows, dotted arrow or black arrowheads indicate bands for CycG1, 6Myc-H2AX, CycG2 and 6Myc proteins, respectively. L: pre-cleared lysate. B: IP products in beads. (**c**) Western blot analyses performed on immunoprecipitates of extracts from HEK-293T cells expressing 6Myc-H2AX to determine whether camptothecin and/or γ-IR treatment influences the association between 6Myc-H2AX and PP2A C. Precipitation by IgG was used as a negative control. Turquoise arrows indicate bands for the putative complex between 6Myc-H2AX and PP2A C (lanes 9 and 15); gray arrows indicate that no such band was detected in lanes 12 and 18. (**d**) Schematic illustrations showing that the complex between 6Myc-H2AX and PP2A C that forms in WT MEFs (left) after camptothecin or γ-IR treatment does not form in the absence of CycG2 (right, G2KO MEFs).

The results described above suggest that H2AX affects the function of the PP2A complex via direct interaction with its B’γ subunit. In western blots of extracts from 6Myc-H2AX–expressing WT MEFs, we observed no band corresponding to the catalytic subunit of PP2A (PP2A C) in the absence of γ-IR (lane 6 of Fig. 4a-i). By contrast, 2 h (red arrow) after 10 Gy γ-IR, immunoprecipitated complex from 6Myc-H2AX–expressing WT MEFs cells had a PP2A C band (lane 13 in Fig. 4a-ii). Notably, this band almost disappeared in 6Myc-H2AX–expressing G2KO MEFs (lane 15 in Fig. 4a-ii), suggesting that CycG2 is required for formation of a complex between H2AX and PP2A C following γ-IR. The band intensities of immunoprecipitates obtained using anti-PP2A C and anti-Myc antibodies were weakened 4 h after γ-IR in both 6Myc-H2AX expressing WT (lane 14) and G2KO (lane 16) MEFs, suggesting that these immune complexes were unstable.

Next, we performed western blot analysis of human embryonic kidney cells harboring large T antigen (HEK-293T) in order to examine the association of CycG1 or CycG2 with H2AX (Fig. 4b). We found that HEK-293T cells expressing 6Myc-H2AX and 3FLAG-tagged CycG1 or CycG2 had a CycG1 band (blue arrowhead; lane 6) or CycG2 band (dotted arrow; lane 14) only in the bead fraction (B; IP products). The intensities of the bands for both CycG1 (lane 5) and CycG2 (lane 13) were stronger than those in pre-cleared lysate (L), suggesting that CycG1 and CycG2 associate with H2AX *in vivo* (Fig. 4b). Notably, the 6Myc-H2AX band almost disappeared 4 h after 10 Gy γ-IR (white arrow, lane 16 in Fig. 4b), decreasing to the background (L) level (dotted arrow, lane 16 in Fig. 4b), suggesting that H2AX was released from CycG2 after DNA repair was completed. By contrast, no such alteration occurred for CycG1, suggesting that the CycG2–H2AX interaction is involved in H2AX-mediated repair of DNA damage other than that induced by γ-IR.

We also investigated whether camptothecin (5 μM) treatment would also influence the complex formation between H2AX and PP2A C. After treatment of WT MEFs with camptothecin or 10 Gy γ-IR (tilted blue arrows in lanes 6 and 9 in Fig. 4c), H2AX immunoprecipitates contained a PP2A C band, but no such band was detected in G2KO MEFs (tilted gray arrows in lanes 15 and 18 in Fig. 4c). Taken together, these results suggest that CycG2 is required for the interaction between H2AX and PP2A C after camptothecin treatment or 10 Gy γ-IR, as illustrated in Fig. 4d.

### Cyclin G1 and Cyclin G2 in cancer patients

**Figure 5:**
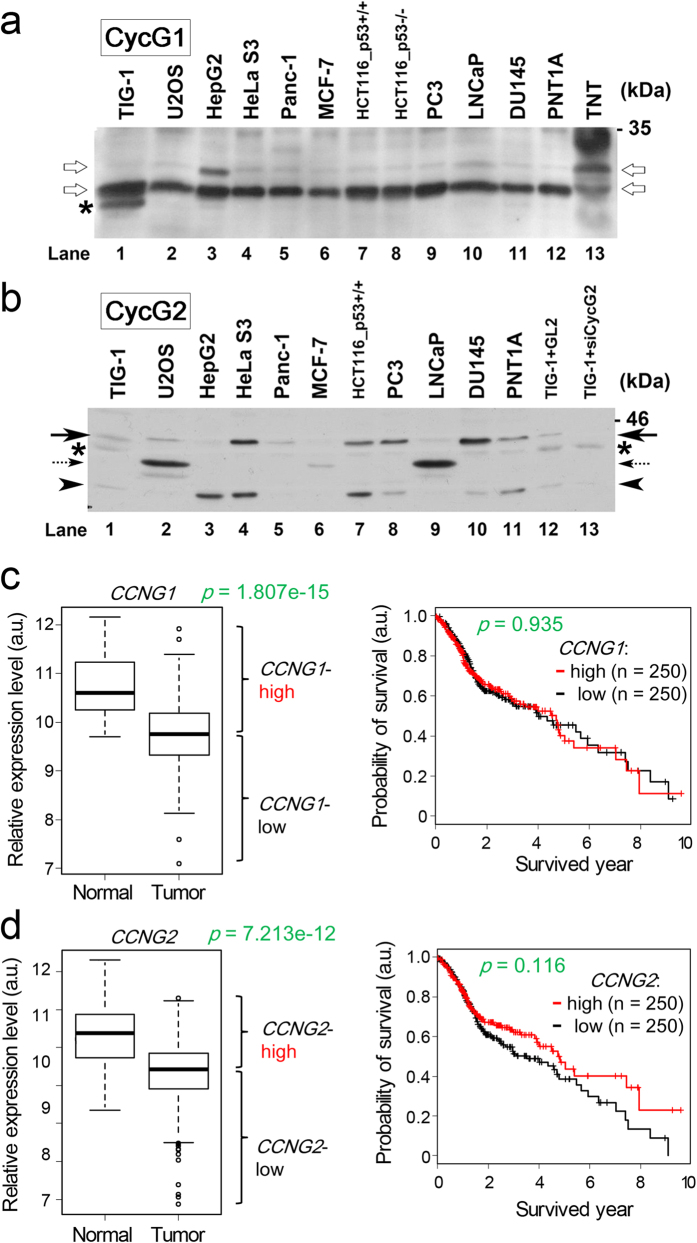
CycG1 and CycG2 expression levels in cancer cell lines and cancer patients. (**a,b**) Western blot analysis of the indicated cell lines using anti-CycG1 (**a**) or anti-CycG2 (**b**) antibodies. The CycG1 band was recognized by the TNT product (white arrows). The CycG2 band was identified by the disappearance of a band (black arrows) following siCycG2-mediated knockdown (lane 13). Arrowhead indicates a band whose intensity was weakened but not abolished by siCycG2-mediated knockdown. Asterisks denote putative non-specific bands. (**c**) Box-and-whisker plots of CycG1 (*CCNG1*) mRNA expression in normal and tumor regions of head and neck cancer patients (i), and Kaplan–Meier survival curves for survival (days) depicted based on TCGA data for *CCNG1* (ii). (**d**) Box-and-whisker plots of CycG2 (*CCNG2*) mRNA expression in normal and tumor regions of head and neck cancer patients (i), and Kaplan–Meier survival curves for survival (days) depicted based on TCGA data for *CCNG2* (ii). Statistical significance of differences between normal and tumor regions was evaluated by Wilcoxon rank-sum test.

Next, to determine whether expression levels of CycG1 and CycG2 are abnormal in cancer, we performed western blot analysis in cancer cell lines. To identify the authentic band for CycG1, we expressed CycG1 protein in a transcription/translation (TNT) system, resulting in two bands near the predicted size of CycG1 (lane 13, white arrows in Fig. 5a). The CycG1 level was almost identical in cancer cell lines (lanes 2–12) and the normal fibroblast strain TIG-1 (lane 1). One exception was the upper band in HepG2 cells (lane 3), in which CycG1 mediates suppression of p53 by Notch3 and modulates the Notch signaling^43^.

By contrast, in some cancer cells, at least two CycG2 bands were detected, and the intensity of the upper band at the predicted size for CycG2 (black arrow) was elevated (lanes 4, 7, 8, 10, and 11 of Fig. 5b) relative to TIG-1 (lane 1). siRNA-mediated knockdown of CycG2 abolished this band in TIG-1 cells (lane 13 in Fig. 5b). The lower band (arrowhead) may not correspond to the CycG2 band because it is smaller than intact CycG2 and siCycG2-mediated knockdown was incomplete. It remains unknown whether the middle bands (dotted arrow) detected in U2OS and LNCaP cells (lanes 2 and 9) represents the CycG2 band. These results suggest that CycG2 expression is abnormal in many cancer cells.

To determine whether the aforementioned abnormal phenotypes in mice and MEFs are reflected in human cancer patients, we visualized relative expression levels of these genes in 8,415 RNA-seq datasets from The Cancer Genome Atlas (TCGA) data portal (https://tcga-data.nci.nih.gov/tcga/). The expression level of CycG1 (*CCNG1*) was reduced in bladder cancer, head and neck cancer, and sarcoma relative to normal solid tissue (Supplementary Fig. S9a), whereas CycG2 (*CCNG2*) expression was reduced in colon cancer, head and neck cancer, and rectal cancer (Supplementary Fig. S9b). Because both *CCNG1* and *CCNG2* were dysregulated in head and neck cancer, we generated Kaplan–Meier curves and estimated the effect of *CCNG1*/*CCNG2* expression status on survival. Little difference was observed with respect to CycG1 (*CCNG1*) expression (Fig. 5c). However, patients with lower-than-median *CCNG2* expression showed a tendency toward poorer clinical prognoses than patients with higher-than-median expression (n = 466), although the difference was not statistically significant (*p* = 0.116) (Fig. 5d). These results suggest that reduced expression of CycG2 is probably associated with cancer pathogenesis.

## Discussion

In this study, we generated G1KO, G1KO, and DKO mice (Supplementary Fig. S1), which were grossly normal and fertile (Supplementary Tables S1 and S2). G2KO MEFs, but not G1KO and DKO MEFs, were resistant to DNA-damaging insults such as camptothecin (Fig. 1a) and γ-IR (Fig. 2 and Supplementary Fig. S4). This is probably because CycG2, but not CycG1, co-localized with γH2AX foci in the nucleus after γ-IR. Indeed, camptothecin treatment generated nuclear foci after IR treatment (Fig. 1b), and γH2AX-mediated DNA repair was delayed in G2KO MEFs relative to WT MEFs (Fig. 1c). Flow cytometry revealed that G2KO MEFs, but not G1KO, DKO or WT MEFs, arrested at G2/M phase of the cell cycle after γ-IR (Fig. 2a and Supplementary Fig. S3). Immunostaining also unveiled that γ-IR caused a delay in S phase in G2KO MEFs after γ-IR, but not in WT MEFs (Fig. 2b), suggesting that S phase delay activated a cell cycle checkpoint, leading to G2/M arrest in G2KO MEFs. Exogenously overexpressed CycG2 rescued this cell cycle arrest, suggesting an essential role for CycG2 in this event (Fig. 2b). Because dephosphorylation of CHK2-pT68 is mediated by the CycG2–B’γ complex, CHK2-pT68 is also likely to have triggered, at least partially, the G2/M arrest of G2KO MEFs^27^. Indeed, CHK2-pT68 band intensity was retained after IR (Fig. 3a). Moreover, G2/M arrest due to inhibition of CycG2–B’γ complex formation also increased the CHK2-pT68 level (Fig. 4). We also found that dephosphorylation of CHK2-pS33/35 and ATM-pS1981 was perturbed only in G2KO MEFs, suggesting that the CycG2–B’γ complex also regulates dephosphorylation of these sites (Fig. 3a). Interestingly, H2AX associated with CycG1, CycG2, and PP2A (Fig. 4), suggesting that γH2AX affects the function of the PP2A complex via direct interaction with its B’γ subunit.

Western analysis revealed that CycG1 expression was almost normal (Fig. 5a), whereas CycG2 expression was abnormal, in various cancer cell lines (Fig. 5b). Kaplan–Meier curves based on TCGA data revealed that head and neck cancer patients with reduced CycG2 (*CCNG2*) expression had poor clinical prognoses (Fig. 5d). We previously showed that drug sensitivity of pancreatic cancer cells is correlated with expression of miR-1246, which targets *CCNG2* mRNA and decreases CycG2 protein levels^41^. Moreover, elevated expression of miR-1246 was correlated with poor prognosis and reduced CycG2 expression. Indeed, the Kaplan–Meier curve for overall survival revealed that reduced expression of CycG2, as determined by IHC, was a consistent indicator of poor prognosis in pancreatic cancer patients^42^. Taken together, these findings suggest that CycG2-deficient tumors have poor prognosis because they are more likely to be resistant to radiotherapy and cytotoxic agents, and consequently to tolerate higher levels of DNA damage. Our findings suggest that low expression of CycG2 may serve as a novel, independent prognostic marker.

## Materials and Methods

All methods were performed in accordance with the relevant guidelines and regulations. Further information can be found in Supplementary information.

### Generation of G1KO, G2KO, and DKO mice

The *Ccng1* gene was targeted by replacing all six exons containing the open reading frame with a PGK-neomycin cassette (*PGK*-*neo*) as a positive selection marker (Supplementary Fig. S1). The targeting construct was generated by cloning the 5′ short arm (*Xba*I–*Sma*I fragment) and the 3′ long arm (*Xba*I–*Xho*I fragment) into a targeting vector, which was injected into ES cells by electroporation. Targeted ES cell clones identified by Southern blot analysis and PCR were microinjected into C57BL/6 blastocysts. Chimeric mice generated by re-implanting the blastocysts into pseudopregnant females were mated with C57BL/6 mice. *Ccng1*^−/−^*Ccng2*^−/−^ DKO mice were generated by mating *Ccng1*^−/−^ single knockout mice with *Ccng2*^−/−^ single knockout mice^27^. Heterozygous offspring were intercrossed to generate homozygous embryos. Gene disruption was confirmed by PCR on genomic DNA from tail snips. Experiments were supported by UNITECH Co., Ltd (Chiba, Japan). Generation of *Ccng2* knockout mice was performed as described previously^27^.

## Ethical permission

All animal experiments were performed with the approval of the Animal Experiments Committee of Osaka University (permission number: BikenA-H24-16-0 and BikenA-H24-17-0).

### Generation of MEFs

MEFs were prepared from embryos of each knockout mouse and maintained in growth medium as described previously^27^. Briefly, mouse embryos were removed from the uterus at embryonic day 12.5–14.5, and a section of the embryo was used for genotyping. Primary MEFs were obtained as described previously^43^. The seeding of trypsinized MEFs onto a 60 mm dish was defined as passage 0, and the first re-plating onto a 100 mm dish was defined as passage 1. MEFs were cultured at 37 °C in 5% CO_2_ in Dulbecco’s modified Eagle’s medium (DMEM) (Sigma-Aldrich, Tokyo, Japan) supplemented with 10% heat-inactivated fetal bovine serum (FBS) (HyClone, SV30014.03), 100 U/mL penicillin G, 100 mg/mL streptomycin sulfate (Nacalai Tesque, Kyoto, Japan), and 50 mM 2-mercaptoethanol (GIBCO). Genotyping and gene expression were performed by genomic PCR and RT-PCR, respectively.

### Antibodies

Antibodies raised against the following proteins were purchased from the indicated commercial sources. Monoclonal antibodies: FLAG, α-tubulin, and CHK2 (Sigma-Aldrich); GAPDH (Fitzgerald Industries International); γH2AX (Millipore); Myc (PL14, MBL); PLK-pT210 (Cell Signaling); p21 and Kip1 (BD Biosciences). Polyclonal antibodies: ATM (Abcam); (BD Biosciences); ATM-pS1981 (Rockland Immunochemicals); CDC2 (Santa Cruz Biotechnology); CycG1 (UBI); CycG2 (custom antibody generated by MBL); H2AX and POLK (ab11175, Abcam); CHK2-pT68, PLK-pT210, PP2A_C, and SOX2 (Cell Signaling); WT1, HOXA2, HOXB4, HOXB7, and HOXB9 (Biorbyt).

### DNA microarray analysis

The microarray data have been deposited in the Gene Expression Omnibus (GEO; www.ncbi.nlm.nih.gov/geo) database (accession number GSE79618).

## Additional Information

**How to cite this article**: Ohno, S. *et al*. Comprehensive phenotypic analysis of knockout mice deficient in cyclin G1 and cyclin G2. *Sci. Rep.* **6**, 39091; doi: 10.1038/srep39091 (2016).

**Publisher's note:** Springer Nature remains neutral with regard to jurisdictional claims in published maps and institutional affiliations.

## Supplementary Material

Supplementary Data
