# Supplementary material for: Comprehensive phenotypic analysis of knockout mice deficient in cyclin G1 and cyclin G2
Source: Sci Rep. 2016 Dec 16;6:39091. doi: 10.1038/srep39091 (PMC5159814; doi:10.1038/srep39091)
Supplement: Supplementary Data [file srep39091-s1.pdf]

Comprehensive phenotypic analysis of knockout mice deficient in cyclin G1 and cyclin G2

Shouichi Ohno, Jun-ichiro Ikeda, Yoko Naito, Daisuke Okuzaki, Towa Sasakura, Kohshiro Fukushima, Yukihiro Nishikawa, Kaori Ota, Yorika Kato, Mian Wang, Kosuke Torigata, Takashi Kasama, Toshihiro Uchihashi, Daisaku Miura, Norikazu Yabuta, Eiichi Morii, and Hiroshi Nojima

Supplemental Results

Loss of CycG1 and CycG2 safeguards against diethylnitrosamine (DEN)-induced hepatocarcinogenesis. The results above suggested that G2KO mice tend to develop resistance to DSB-related carcinogens. To explore if G2KO mice can become resistant to other carcinogens, we examined DEN-induced hepatocarcinogenesis in G2KO mice, because G1KO mice were previously reported to be resistant to DEN5. To examine whether G2KO and DKO mice are also resistant to DEN, we induced liver tumors with a single DEN injection in 14-day-old mice, and determined the presence of hepatic tumors 36 weeks after the DEN treatment. We found that the number and size of tumors and liver weight were conspicuously reduced, to similar extents, in all three mutants at the 36 week time point, when all WT animals had developed large liver tumors (Supplementary Fig. S6a, S6b). This was probably due to the absence of large tumors in these mutant mice because the frequency of tumors larger than 2.5 mm was reduced in G1KO, G2KO, and DKO mice (Supplementary Fig. S6c) at levels corresponding to liver weight (Supplementary Fig. S6b). The similarly low levels of carcinogenesis among these three genotypes suggest that both CycG1 and CycG2 are resistant to DEN by acting in the same signaling pathway leading to DEN-induced hepatocarcinogenesis.

Histological analysis revealed nodular lesions of varied sizes in almost normal background liver (Supplementary Fig. S7). Tumor cells exhibited a trabecular architecture resembling the normal sinusoids of the liver, and hepatic cells had central round nuclei and eosinophilic cytoplasm. Steatosis was detected in several regions. These findings indicated that the tumors were well to moderately differentiated hepatocellular carcinoma (HCC). Histologically, there was little difference among the four genotypes of mice. Active caspase-3 was barely detectable by immunohistochemistry in DEN-induced HCC tissues of all genotypes. Since decreased tumor susceptibility to DEN-induced hepatocarcinogenesis in G1KO mice is attributed to a negative feedback system constituted by cyclin G1 and

MDM2-pT2166, we suggest that reduced susceptibility of G2KO mice to tumorigenesis could also be due to changes in this system (Fig. 3b). Because the molecular mechanism that regulates the DEN-induced hepatocarcinogenesis is very complex, we will refrain from proposing a hypothesis to explain these observations at this time. Data obtained by DNA microarray analysis of G1KO, G2KO, and DKO MEFs (see Supplementary Fig. S8) may suggest a direction of our forthcoming research. Future experiments in our group will focus on the involvement of CycG1 and/or CycG2 in this phenomenon.

DNA microarray analysis of G1KO, G2KO, and DKO MEFs. To determine whether the aforementioned phenotypes are correlated with the expression levels of any specific genes, we performed DNA microarray analysis on G1KO, G2KO, and DKO MEFs. Samples for RNA purification were obtained during logarithmic growth phase. Scatter plots (Supplementary Fig. S8a) and fold-change comparisons (Supplementary Table S3) indicated that mRNA levels of some genes were up- or down-regulated in all three strains of mutant MEFs. Notably, the fold-change values (Supplementary Table S3) were much larger in DKO MEFs than in G1KO or G2KO MEFs, suggesting that loss of CycG1 and CycG2 caused uncontrolled expression of genes mutually regulated by both proteins, and further suggest that the functions of CycG1 and CycG2 overlap and complement each other. Moreover, these findings suggest that the genes up- or down-regulated in the DKO MEFs are actually controlled by CycG1 and/or CycG2.

Interestingly, mRNA levels of asporin/Aspn, a small leucine-rich proteoglycan, were down-regulated in G2KO and DKO MEFs (green font in Supplementary Table S3). Because asporin is predominantly expressed in cancer-associated fibroblasts (CAFs), and promotes co-invasion of CAFs and cancer cells in scirrhous gastric cancer41, down-regulation of asporin may explain at least in part why G2KO and DKO mice were resistant to DEN-induced hepatocarcinogenesis (Supplementary Fig. S6). Up-regulation of X-inactive specific transcript (Xist), an RNA gene on the X chromosome that influences the X inactivation process42, in G1KO and G2KO MEFs is due to the female origin of these MEFs and is therefore very likely to be unrelated to their phenotypes. Notably, mRNA levels of several transcription factor genes were upregulated in G2KO (En2, Foxa2, Sox2, and Sox6) and DKO (WT1, Foxa2, Hoxb2, Hoxb4, Hoxb7, and Hoxb9) MEFs.

To determine whether these differences in mRNA levels are reflected in the protein levels, we performed western blot analysis on several transcription factors (WT1, Hoxa2, Hoxb2, Hoxb7, and Hoxb9). As expected from the mRNA levels (Supplementary Fig. S8a), the WT1 protein level was significantly higher in DKO MEFs than in WT, G1KO, or G2KO MEFs (Supplementary Fig. S8b). The Hoxa2 level was reduced and Hoxb9 level was slightly augmented only in G2KO MEFs. By contrast, the protein levels of other tested transcription factors were not significantly altered (Supplementary Fig. S8b), suggesting that depletion of

CycG1 and CycG2 had little impact on their expression. It remains unclear how these differences might influence the aforementioned phenotypes of these MEFs, and we intend to address this issue in a future study. Taken together, these results suggest that CycG1 and CycG2 mutually regulate the mRNA and protein levels of WT1.

Supplemental Materials and methods

Cell culture and transfection. Human osteosarcoma U2OS cells were maintained in DMEM, Sigma-Aldrich, D5796) supplemented with 10% FBS (HyClone, SV30014.03) with 100 U/ml penicillin/100 μg/ml streptomycin (Nacalai Tesque, #26253-84). For culture of U2OS/pTet-On_Advanced and U2OS/Myc-CycG2 cells, 0.8 mg/μl G418 Disulfate Aqueous Solution (Nacalai Tesque, #16513-26) was added to the medium. For culture of U2OS/pTet-On_Myc-vector and U2OS/pTet-On_Myc-ELAS2 cells, hygromycin B (Clontech, #631309) was added to the medium at 0.2 mg/μl.

Genomic PCR. Genomic DNA purification and PCR was performed as described previously.[27] In brief, for genomic PCR on mouse genome DNA, ExTaq polymerase (Takara, Shiga, Japan) was used with the PCRx Enhancer System (Invitrogen, Carlsbad, CA, USA) under the following conditions: a pre-heating step (94°C for 3 min), 40 reaction cycles (94°C for 30 sec, 58°C for 30 sec, and 72°C for 1 min and 40 sec), and a final elongation step (72°C for 6 min).

RT-PCR. RNA preparation and RT-PCR were performed as described previously.[27] In brief, total RNA was extracted from MEFs using the RNeasy kit (QIAGEN, Tokyo), and cDNA was synthesized from 3 μg of RNA using the High-Capacity cDNA Archive Kit (Applied Biosystems, Foster City, CA, USA). PCR primer pairs were as follows: *Ccng1*: forward, 5’-ACCAATCTGGCTAATAGAGGCCAGACC-3’, and reverse, 5’-ATGACTCAGTGTGGCACGGTGCTTAGC-3’; *Ccng2*: forward, 5’-GTGTCCAGGATTGAGAAATGCC-3’, and reverse, 5’-GGCACAAGGCTAATACAGATGG-3’; *Gapdh*: forward, 5’ -TCACCATCTTCCAGGAGCGAG-3’, and reverse, 5’-GCTGTAGCCGTATTCATTGTC-3’. PCR was performed under the following conditions: a pre-heating step (94°C for 2 min) followed by 30 reaction cycles (94°C for 30 sec, 55°C for 30 sec, and 72°C for 30 sec), and a final elongation step (72°C for 5 min). PCR products were separated by agarose gel electrophoresis and visualized by ethidium bromide staining.

Preparation of Tet-On® Advanced Inducible cell lines. For preparation of tetracycline-inducible U2OS cells (hereafter, U2OS/Tet-On cells), U2OS cells were

transfected with the pTet-On Advanced plasmid vector (Clontech) and incubated in DMEM supplemented with 10% FBS, penicillin/streptomycin, and 0.8 mg/ml G418. Several single colonies were selected for further examination.

Vector pTRET3-6Myc was constructed by inserting a multiple cloning site linker containing the 6Myc-tag (*Bam*HI-*Hind*III-*Cla*I-6Myc-*Asc*I-*Eco*RV-*Not*I-*Sal*I) into the *Bam*HI and *Sal*I sites of pTRE-Tight (Clontech). U2OS/Tet-On cells were co-transfected with each of the pTRET3-6Myc plasmids (including CycG1, CycG2, and empty vector) and the Linear Hygromycin Marker (Clontech) using the Lipofectamine PLUS reagent (Invitrogen). Transfected cells were diluted and selected in culture medium containing hygromycin (0.2 mg/ml). Single colonies were isolated, and positive clones were confirmed by examining the expression patterns in the presence or absence of doxycycline (Dox; 1 μg/ml).

DNA microarray analysis. Microarray analyses were performed as dye-swapped experiments for comparisons between WT, G1KO, and G2KO MEFs, and as single-color experiments for comparison between WT and DKO MEFs. Total RNAs obtained from WT, G1KO, G2KO, and DKO MEFs during logarithmic growth phase were independently reverse-transcribed using oligo-dT primers containing the T7 RNA polymerase promoter sequence to generate cDNAs and MMLV-RTase. These were then subjected to *in vitro* transcription using T7 RNA polymerase to label the cRNAs with Cy3-CTP using a Fluorescent Linear Amplification Kit (Agilent Technologies), or Cy5-CTP (Amersham Pharmacia Biotech, Piscataway, NJ) in the case of dye-swapped experiments. Purified Cy3 (or Cy5)-labeled cRNAs (825 ng) from individual MEFs were hybridized on the microarrays. Washing, scanning, and gene analysis with Agilent's Whole Mouse Genome Microarray (4×44K; G4122F, 4x44Kv2; G4846A), which contains 27,204 RefSeq genes, were conducted according to the manufacturer's protocol (Agilent Technologies). Agilent Feature Extraction software (v. 9.5.1) was used to assess spot quality and extract feature intensity statistics. The Subio Platform and Subio Basic Plug-in (v1.19; Subio Inc., Aichi, Japan) were used to calculate between-sample fold change. When large numbers of genes were automatically classified by the software into a signaling pathway heatmap, the genes whose mRNA levels were significantly different between WT, G1KO, G2KO, and DKO MEFs were selected.

Histological analysis. Samples (22 total) were obtained from patients diagnosed with DEN-induced hepatocellular carcinoma (HCC). Histological specimens were fixed in 10% formalin and routinely processed for paraffin embedding. The specimens were sectioned at 4 μm thickness and subjected to hematoxylin/eosin and immunohistochemical staining.

Immunohistochemistry for active caspase-3. Active caspase-3 expression was examined immunohistochemically using anti-active Caspase-3 antibody (Abcam, Cambridge,

UK) to detect apoptotic cells. Antigen retrieval was performed with a Pascal pressurized heating chamber (Dako). The sections were incubated with anti-active caspase-3 (×25) antibody and treated with the ChemMate EnVision kit (Dako). Diaminobenzidine (Dako) was used as the chromogen. As a negative control, staining was performed in the absence of primary antibody. When active caspase-3 signals were detected in a tumor, they were interpreted as representing apoptotic cells. Staining was evaluated independently by two pathologists (J.I. and E.M.).

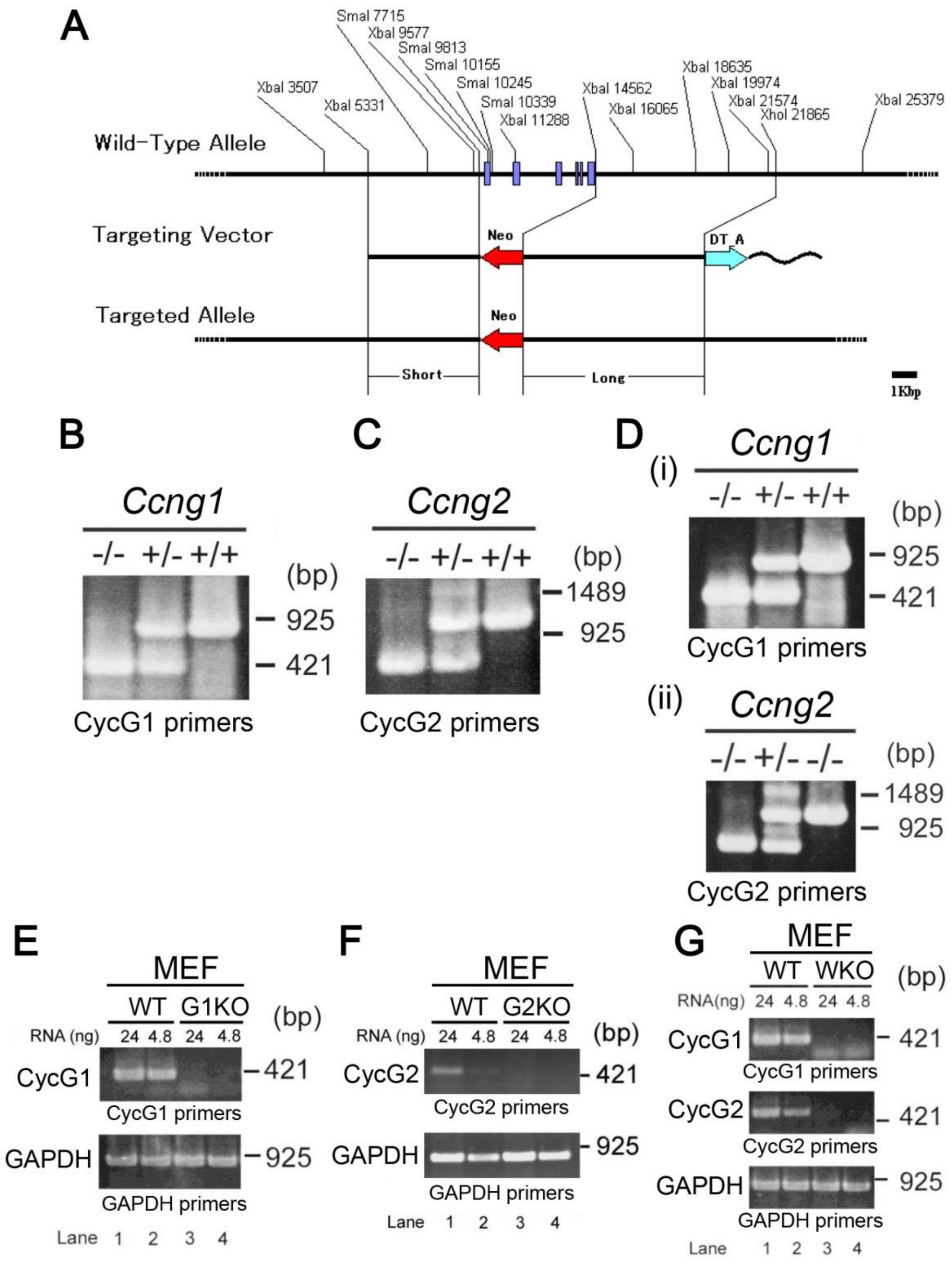

Figure S1. Generation of G1KO and DKO mice. (a) Schematic depiction of the targeting construct and mutant allele used to generate the *G1KO* null knockout mice. Exons are indicated by purple boxes. Neomycin (Neo) and diphtheria toxin A (DT_A) gene cassettes are indicated by red and light blue arrow-shaped boxes, respectively. **(b)** Genotype analysis by PCR using genomic DNA from mice generated by mating *G1KO* heterozygotes. **(c)** Genotype analysis by PCR using genomic DNA from mice generated by mating *G2KO* heterozygotes. **(d)** Genotype analysis by PCR using genomic DNA from mice generated by mating compound heterozygous *Ccng1*$^{+/-}$ *Ccng2*$^{+/-}$ mutant mice, using CycG1 primers **(b, d-i)** and CycG2 primers **(c, d-ii)**. The *G1KO* (i) and *G2KO* alleles (ii) were confirmed as described in **(b)** and **(c)**, respectively. Genotype analysis of *G1KO* (E), *G2KO* (F), and *DKO* MEFs **(g)** by RT-PCR of mRNA isolated from relevant MEFs using CycG1 primers **(e, g)** and CycG2 primers **(f,g)**. The equivalent amount of RNA samples loaded in each lane (24 or 4.8 ng) was used for analysis of *WT* and *G1KO* MEFs. *Gapdh* was used as a positive control.

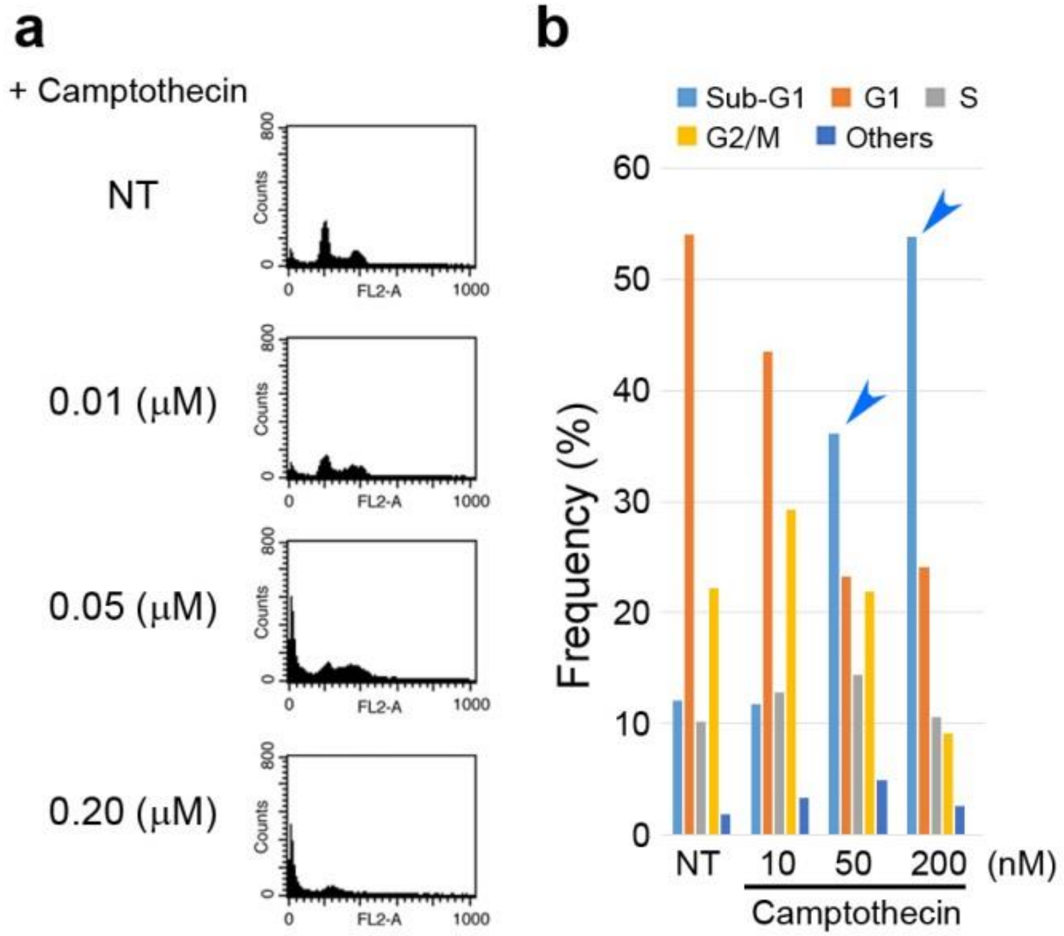

Figure S2. Flow cytometry analysis of SAS human tongue carcinoma cells after camptothecin treatment. (a) SAS cells were subjected to flow cytometry analysis 48 h after treatment with 10, 50, and 200 nM of camptothecin. **(b)** Bar graphs to show frequencies of the indicated populations; sub-G1, G1, S, G2/M, and others. NT, non-treated.

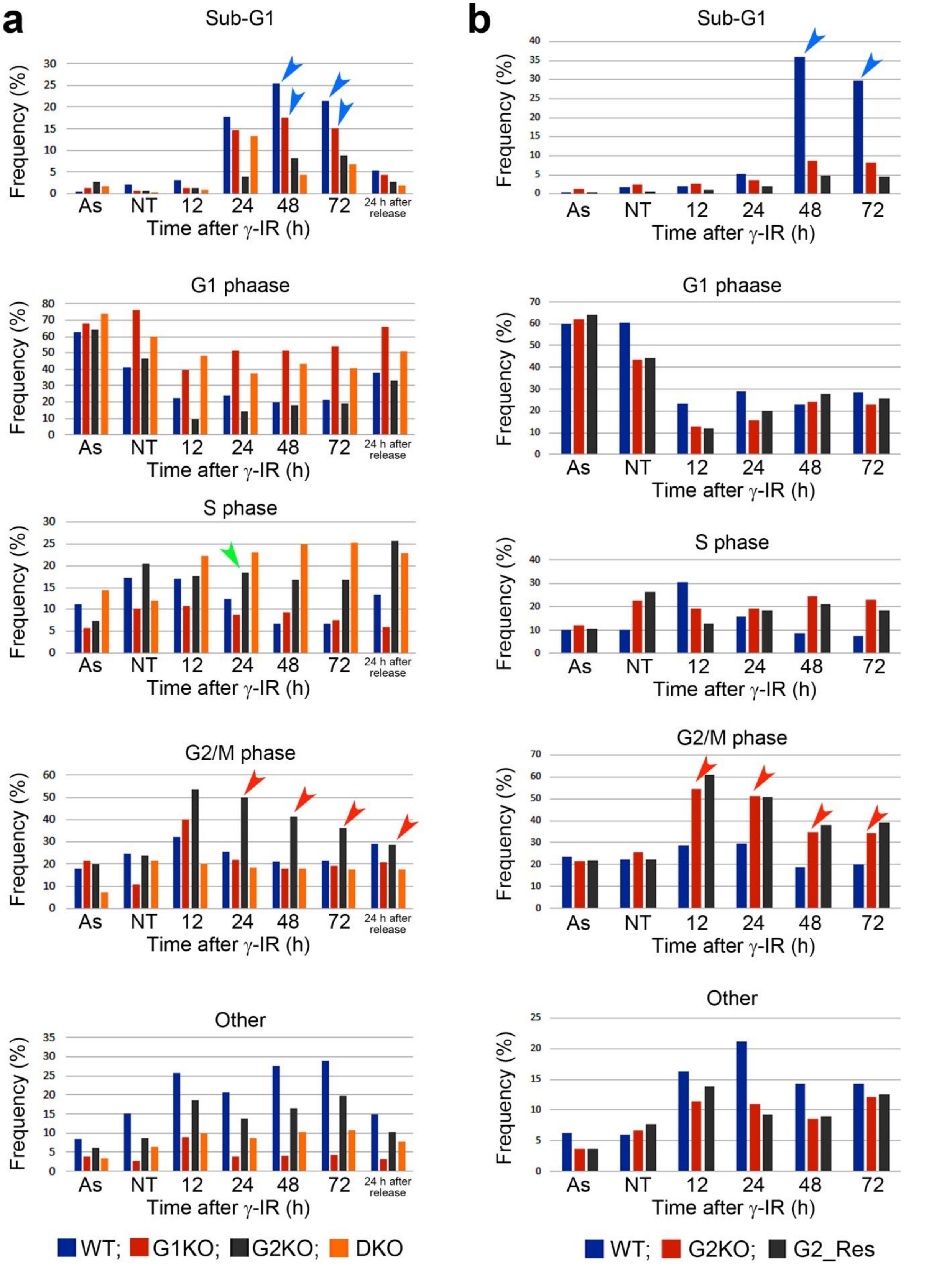

Figure S3. Bar graphs showing frequencies of the indicated populations; sub-G1, G1, S, G2/M, and others. Data were adopted from Fig. 2a **(a)** and Fig. 2c **(b)**. Blue and red arrowheads correspond to those of Fig. 2a and Fig. 2b. Purple arrowhead denotes the S phase peak at 24 h after γ-IR treatment in G2MO MEFs. As, asynchronous growth. NT, non-treated.

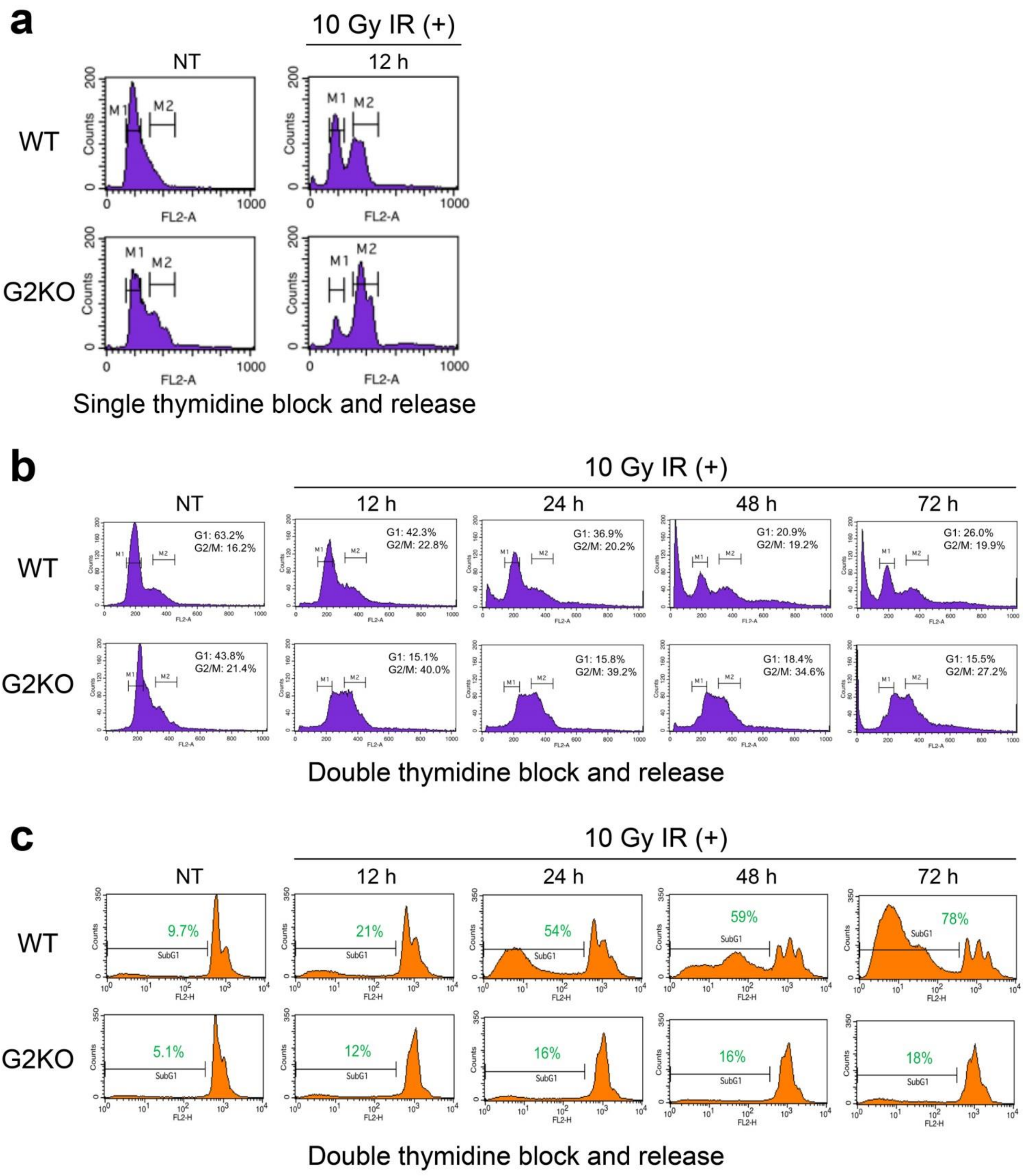

Figure S4. Flow cytometry analysis of WT and G2KO MEFs following 10 Gy γ-IR treatment. (a–c) G2/M arrest phenotype of G2KO MEFs was observed using two distinct synchronization methods, single thymidine block **(a)** and double thymidine block **(b, c)**. NT, non-treated.

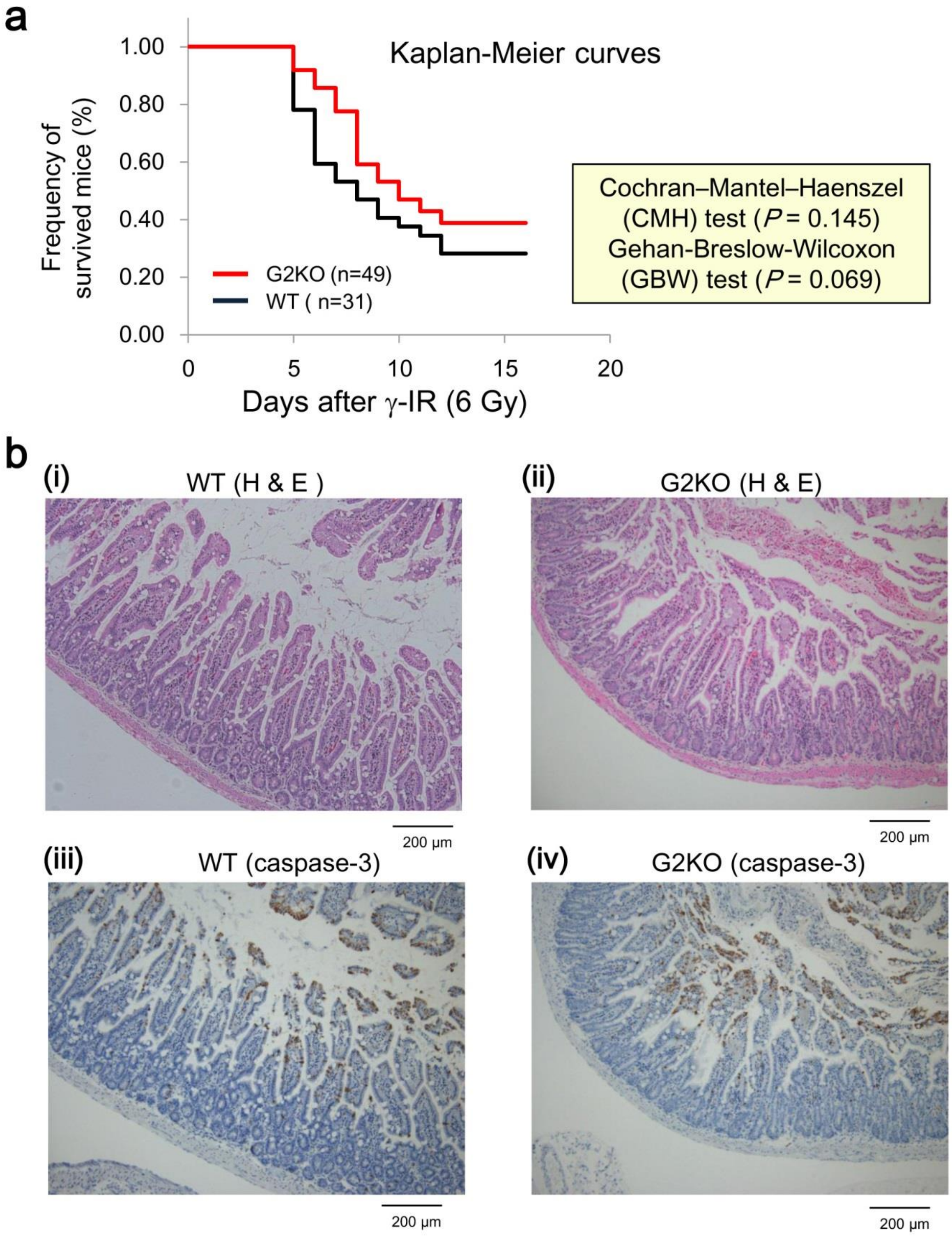

Figure S5. G2KO mice are resistant to γ-IR treatment. (a) Survival curves for WT and G2KO mice for 13 days following γ-IR treatments. The survival frequency of 17–19-week-old mice irradiated with 6 Gy γ-ray was determined by observing them daily for 13 days. **(b)** Histology of small intestines from WT (i) and G2KO (ii) mice, stained with hematoxylin and eosin (H & E; x100). (iii, iv) Immunohistochemistry (IHC) by active caspase-3 for WT (iii) and G2KO (iv) mice (x100). Positive staining for active caspase-3 was detected in epithelial cells of both WT (iii) and G2KO (iv) mice. However, a small amount of positive tumor staining for active caspase-3 was detected in tumor cells of WT (iii) and G2KO (iv) mice. Bar, 200 μm.

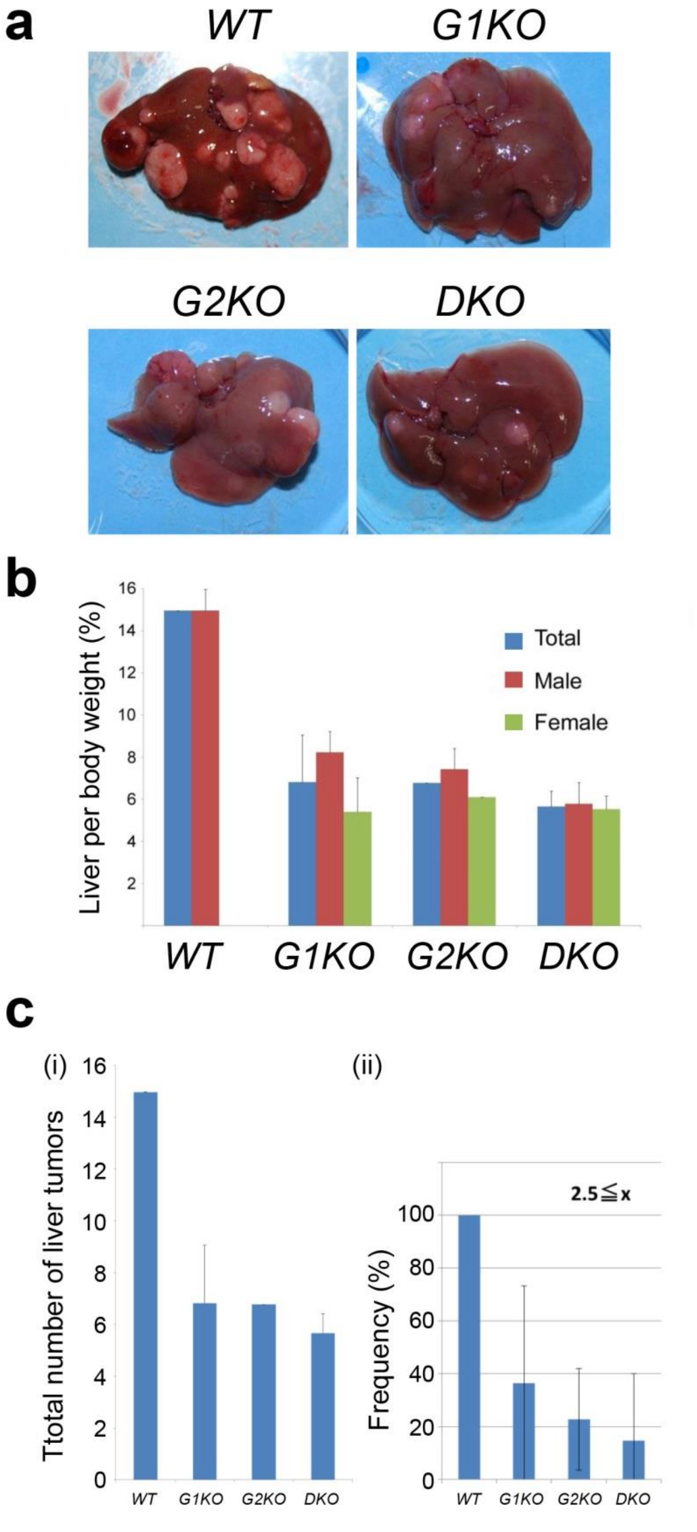

Figure S6. G1KO, G2KO, and DKO mice are resistant to DEN-induced hepatocarcinogenesis. (**a**) Typical images of livers from WT, G1KO, G2KO, and DKO mice harboring hepatocellular carcinomas. Mice were injected intraperitoneally with 10 μg of DEN per body weight (g), and sacrificed 36 weeks after treatment. **(b)** Bar graphs of liver/body weight of WT, G1KO, G2KO, and DKO mice 36 weeks after the DEN treatment. **(c)** Total number of liver tumors (i) and frequency of animals with liver tumors greater than 2.5 mm in diameter (ii).

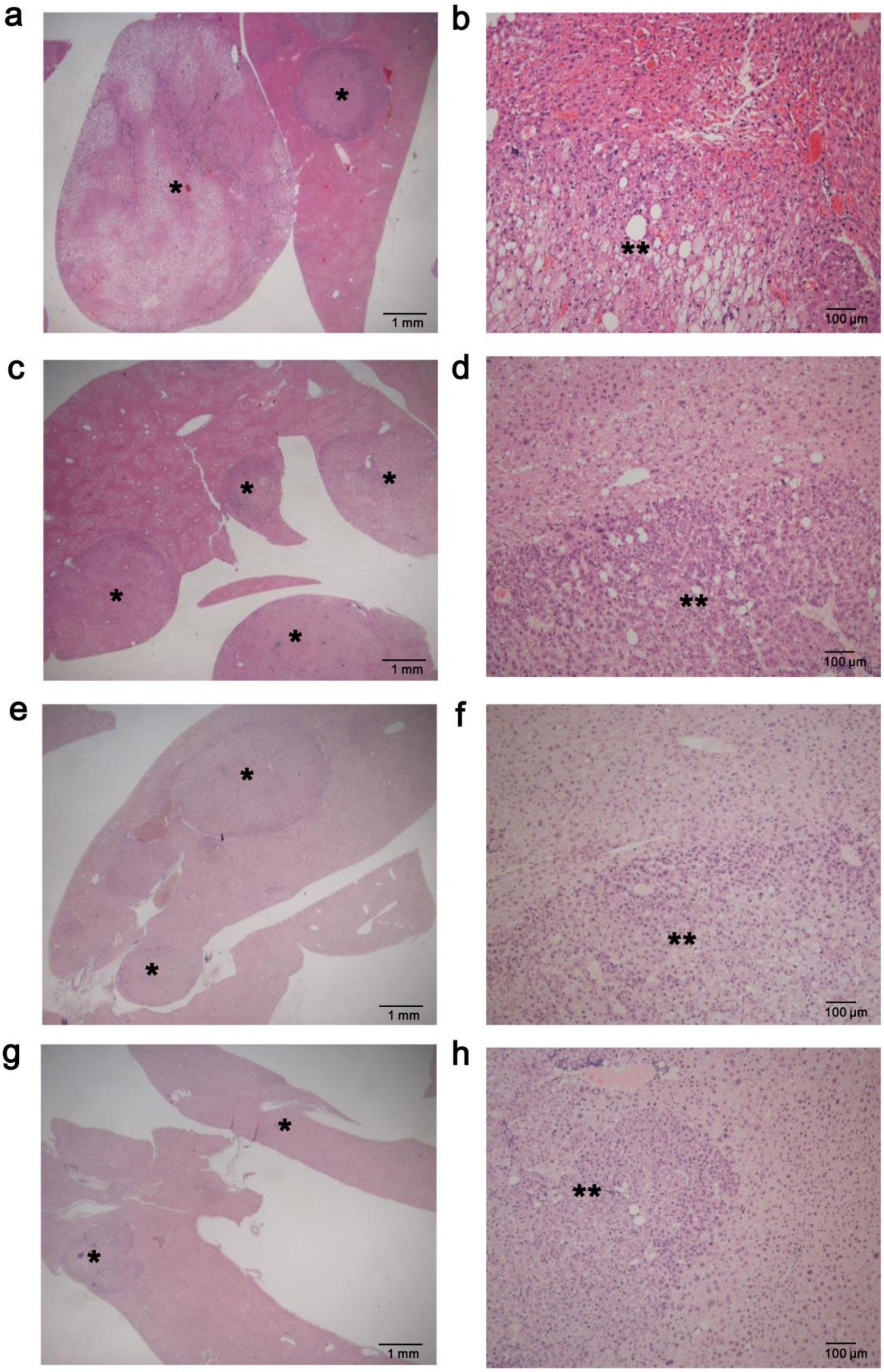

Figure S7. Histological analysis of liver tumors obtained from DEN-treated WT, G1KO, G2KO, and DKO mice. (a-h) H & E staining of livers from WT **(a, b)**, G1KO **(c, d)**, G2KO **(e, f),** and DKO **(g, h)** mice 36 weeks after DEN treatment (**a, c, e, g**; x12.5; **b, d, f, h**; x100). At low magnification, nodular lesions (*) are visible in almost normal background liver. At high magnification, tumor cells (**) exhibit a trabecular architecture resembling the normal sinusoids of the liver. These cells possessed central round nuclei and eosinophilic cytoplasm. Some tumor cells exhibited steatosis. Bar, 1 mm or 100 μm.

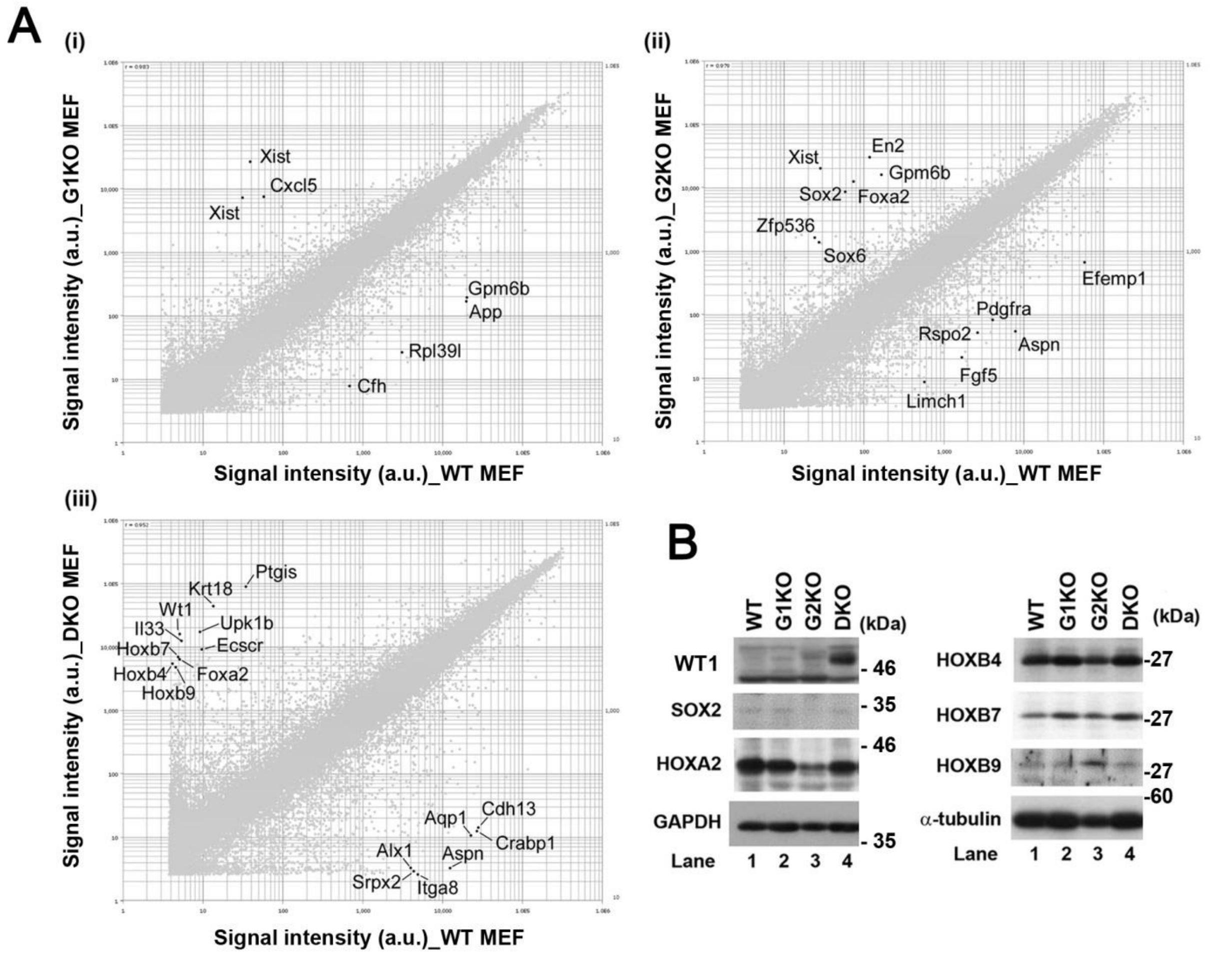

Figure S8. Expression profiling of G1KO, G2KO, and DKO MEFs based on DNA microarray and western analyses. (a) Scatter plots of DNA microarray data for G1KO, G2KO, and DKO MEFs versus WT MEFs. The y-axis shows the log value of hybridization signal intensity (arbitrary unit; a.u.) obtained from microarray data for G1KO (i), G2KO (ii), and DKO (iii) MEFs. The x-axis shows the log value of signal intensity (a.u.) for WT MEFs. Conspicuously up- or down-regulated genes are plotted. **(b)** Western blot for typical transcription factors (WT1, Hoxa2, Hoxb2, Hoxb7, and Hoxb9) on cell extracts obtained from logarithmically growing WT, G1KO, G2KO, and DKO MEFs. GAPDH was used as a loading control.

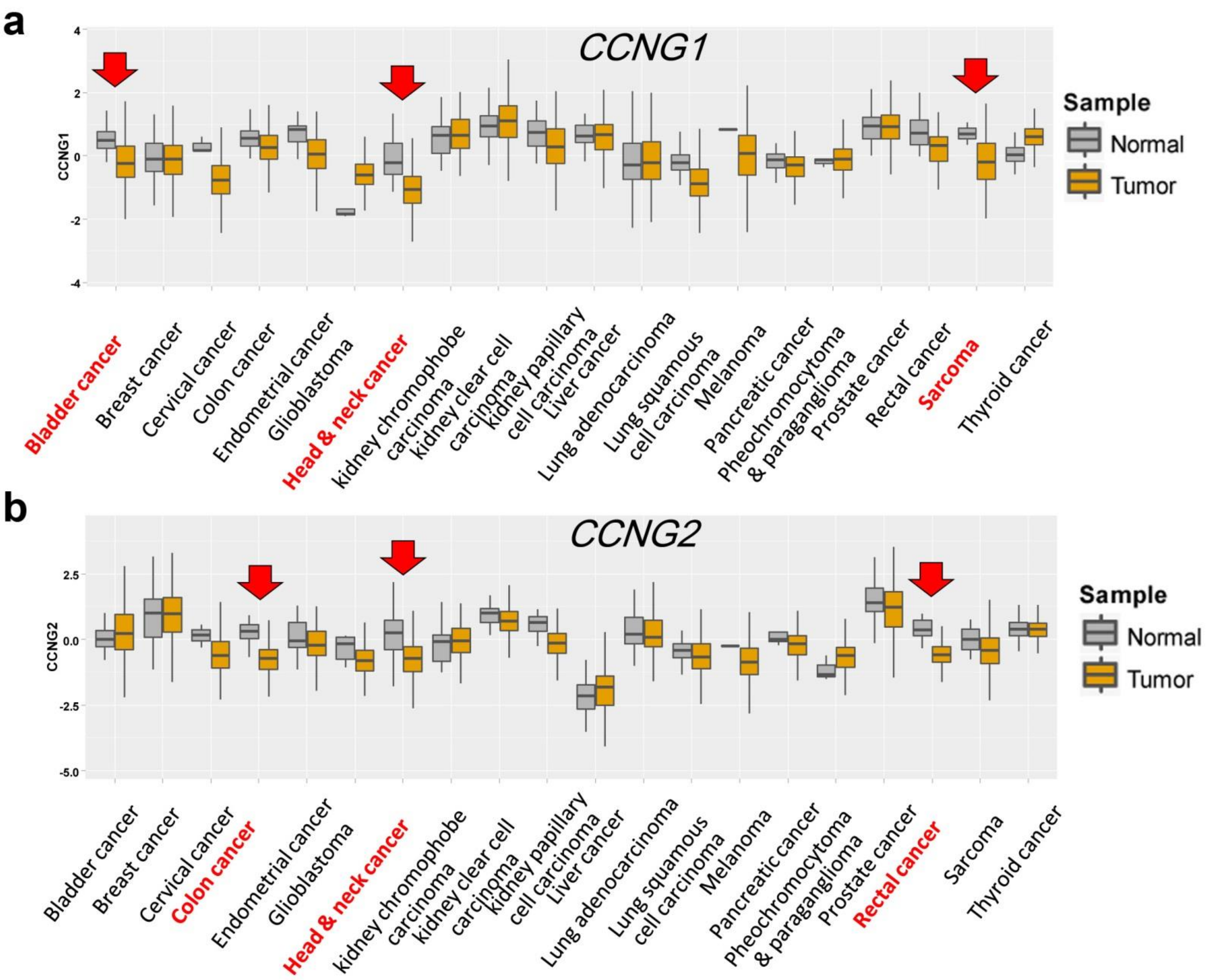

Figure S9. CycG1 (*CCNG1*) or CycG2 (*CCNG2*) is down-regulated in some types of cancers. **(a, b)** Box-and-whisker plots of mRNA expression of *CCNG1* **(a)** and *CCNG2* **(b)** in patients with various types of cancer whose box and names are highlighted by red font and red arrows, respectively. The mRNA expression data for *CCNG1* and *CCNG2* from various cancers and normal tissue samples were obtained from TCGA pan-cancer cohorts. Cancers with at least one normal sample were analyzed and visualized.

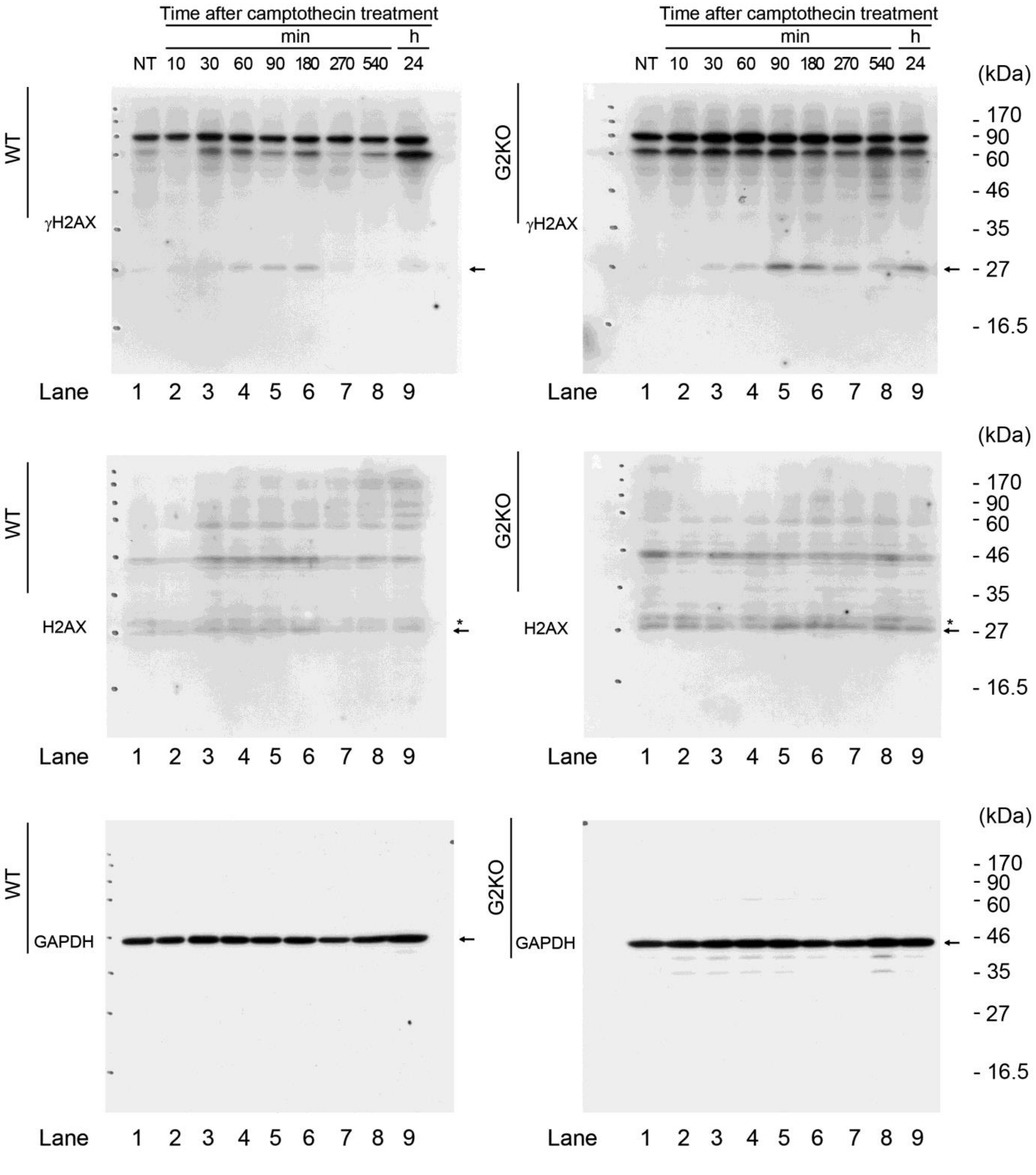

Figure S10. Images of non-cropped western blots. Panels presented in Figure 1c were cropped from these images.

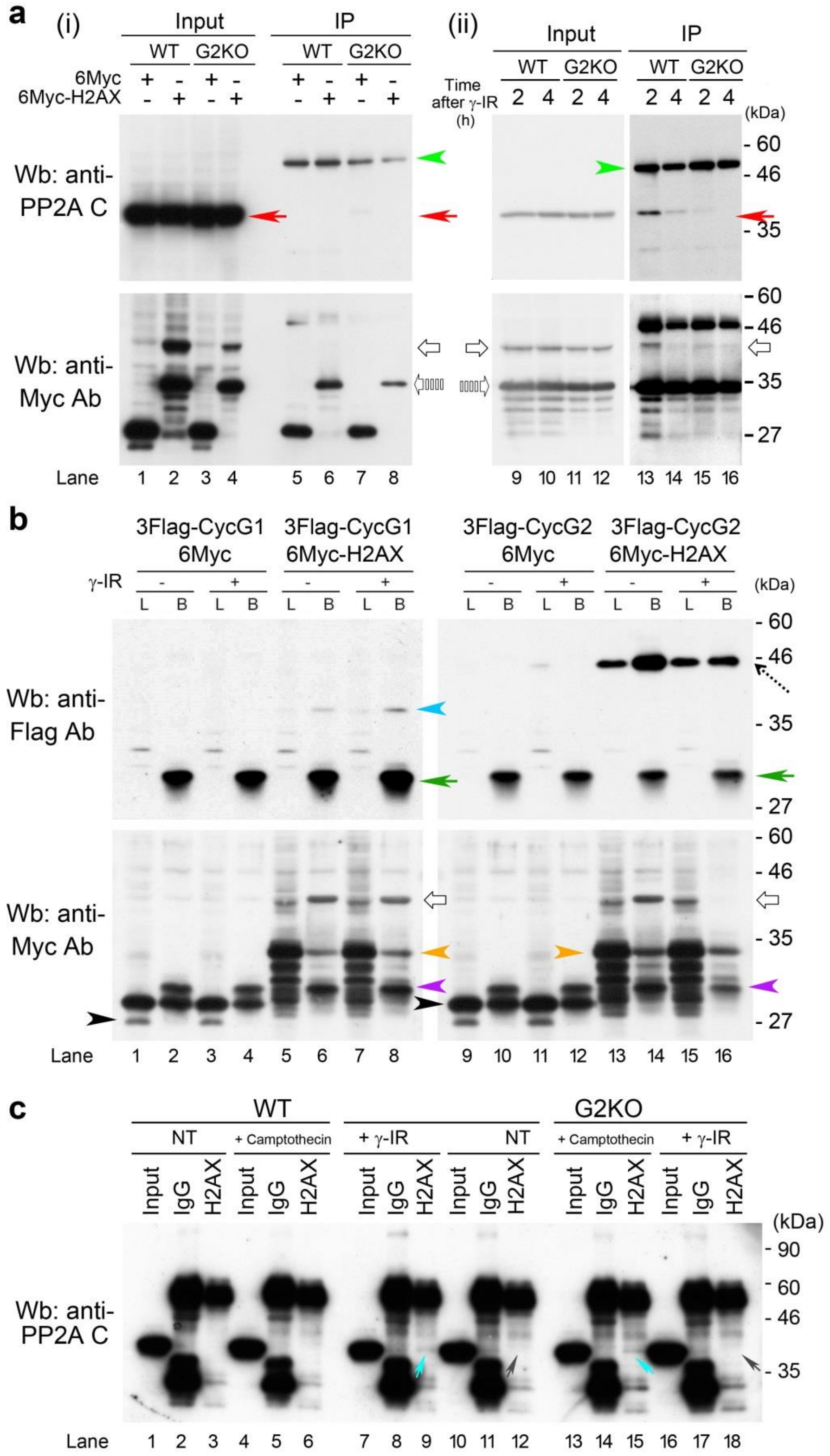

Figure S11. Images of non-cropped western blots. Panels presented in Figure 4 were cropped from these images. Orange arrowheads, black arrowheads or purple arrowheads in (b) indicate bands for putative degradation product of 6Myc-H2AX, 6Myc protein or putative cross-reaction product of green arrow band with anti-Myc antibody, respectively.

Table S1A. Number of male or female mouse in the offspring from *CCNG1* heterozygous intercrosses.

(Total number)	*Ccng1* +/+	*Ccng1* +/-	*Ccng1* -/-
Male (12)	3	5	4
Female (12)	3	5	4
Total (24)	6	10	8

Table S1B. Number of genotypes in the offspring from *CCNG2* heterozygous intercrosses.

(Total number)	*Ccng2* +/+	*Ccng2* +/-	*Ccng2* -/-
Male (74)	20	34	20
Female (66)	16	39	11
Total (140)	36	73	31

Table S1C. Number of genotypes in the offspring from $CCNG1^{+/-};CCNG2^{+/-}$ intercrosses. Numbers in the parentheses indicate the gender ratio (male: female).

(Total number)	*Ccng2* +/+	*Ccng2* +/-	*Ccng2* -/-
CCNG1 +/+	27 (15:12)	27 (11: 16)	9 (3: 6)
CCNG1 +/-	54 (27: 27)	50 (21: 29)	8 (1: 7)
CCNG1 -/-	17 (7: 10)	18 (9: 9)	4 (3: 1)

Table S1D. Number of male or female mouse in the offspring born after intercrosses between $CCNG1^{-/-}CCNG2^{-/-}$ double knockout mice.

(Total number)	*Ccng1, Ccng2* -/-, -/-
Male	76
Female	61
Total	137

Table S2. Occurrence of spontaneous tumors in *CCNG1$^{-/-}$;CCNG2$^{-/-}$*KO mice at 18-30 months.

	WT*		G1KO		G2KO		WKO	
	Male	Female	Male	Female	Male	Female	Male	Female
Undifferentiated carcinoma	0	1	0	0	0	0	0	0
Liver carcinoma	0	0	0	0	0	0	0	1
Liver hemangioma	3	2	0	0	0	0	0	0
Lymphoma	0	1	0	0	0	0	0	0
Fibril formation	0	1	0	0	0	0	0	0
Leukemia	0	0	0	0	0	0	1	0
Ovarian carcinoma	0	0	0	0	0	0	0	1
Lymphoma+hepatocarcinoma	0	1	0	0	0	0	0	0
Lymphoma＋lung carcinoma	0	0	1	1	0	0	0	0
Leukemia + hepatocarcinoma	0	0	1	0	0	0	0	0
Total	3/38(7.9%)	6/44(13.6%)	2/9(22.2%)	1/9(11.1%)	0/3(0%)	0/1(0%)	1/6(16.7%)	2/9(22.2%)

Table S3. List of up-regulated (red font) or down-regulated (green font) genes in G1KO, G2KO and DKO MEFs compared to WT MEFs as revealed by DNA microarray analysis.

G1KO

Gene Symbol	Fold Change
Xist	696.2
Xist	235.4
Ccng1	-196.4
Cxcl5	136.0
App	-117.6
Rpl39l	-117.5
Cfh	-106.0
Gpm6a	-103.7
D3Bwg0562e	96.8
Lrrn4	-75.9
Ddx4	-71.3
Diap2	55.1
Akr1c14	-53.0
Mal2	-49.9
Aldh1a1	-49.2
Upk1b	-47.6
Scel	-45.4
9930013L23Rik	-43.8
Epha3	42.3
Gpm6a	-42.1
Vnn1	-41.4
Ccng1	-39.3
Ms4a3	-38.4
Sfrp2	37.2
2610018G03Rik	-31.6

G2KO

Gene Symbol	Fold Change
Xist	708.8
En2	258.6
Foxa2	174.1
Sox2	147.8
Aspn	-144.3
Limch1	-96.9
Gpm6b	96.6
Efemp1	-87.6
Fgf5	-79.4
Zfp536	67.6
Sox6	51.4
Rspo2	-50.9
Pdgfra	-50.6
Clmp	-46.4
Dnm3os	-44.7
Col11a1	-41.8
Cacna1g	-40.6
Lox	-39.7
Igsf10	-38.9
4833427G06Rik	38.6
Kcnab1	38.1
Sparcl1	-37.8
Cdkn2b	-37.8
Fgf7	-34.3
Sfrp2	-33.5
Col6a3	-33.5
Esm1	-32.5
Slc1a3	-32.4
Chrdl1	-31.7
Ptgs1	-31.6
Dkk2	-30.7

DKO

Gene Symbol	Fold Change
Aspn	-3533.5
Krt18	3491.1
Wt1	3342.7
Ptgis	2780.1
Il33	2475.7
Upk1b	2020.0
Cdh13	-1996.0
Aqp1	-1964.6
Crabp1	-1831.5
Itga8	-1757.4
Hoxb7	1519.5
Hoxb4	1405.2
Srpx2	-1386.9
Foxa2	1341.6
Alx1	-1127.3
Hoxb9	1120.7
Ecscr	1013.1
Grem2	-931.0
Col6a3	-924.2
Ces2g	920.9
Gata6	919.9
Mgst1	887.6
Chrdl1	-871.5
Kctd15	-864.1
Mgst1	857.5
Hoxb2	694.6
Dkk3	-679.7
Acp5	671.3
Crlf1	-652.8
Mcpt8	620.4
